# Supplementary material for: Sociodemographic and lifestyle predictors of incident hospital admissions with multimorbidity in a general population, 1999–2019: the EPIC-Norfolk cohort
Source: BMJ Open. 2020 Sep 22;10(9):e042115. doi: 10.1136/bmjopen-2020-042115 (PMC7509968; doi:10.1136/bmjopen-2020-042115)
Supplement: Supplementary data [file bmjopen-2020-042115supp001.pdf]

Sociodemographic and lifestyle predictors of incident hospital admissions with multimorbidity in a general population 1999–2019: the EPIC-Norfolk cohort

Supplementary material

Supplementary Table S1 | Charlson Comorbidity Index, ICD-10 codes and weighting

Supplementary Table S2 | Descriptive characteristics at TP2 in 9814 men and women aged 48–92 by 10-year Charlson Comorbidity Index, 2009–2019

Supplementary Table S3 | Multivariable logistic regression of risk factors for Charlson 5-year and 10-year hospital admissions with multimorbidity at TP2 in 9814 men and women

Supplementary Table S4 | Multivariable logistic regression of risk factors excluding participants with prevalent CVD, cancer or diabetes at TP2 for Charlson 5-year and 10-year hospital admissions with multimorbidity at TP2 in 8185 men and women

Supplementary Table S1 | Charlson Comorbidity Index, ICD-10 codes and weighting

	ICD-10 codes
Myocardial infarction	I21.x, I22.x, I25.2
Congestive heart failure	I09.9, I11.0, I13.0, I13.2, I25.5, I42.0, I42.5–I42.9, I43.x, I50.x, P29.0
Peripheral vascular disease	I70.x, I71.x, I73.1, I73.8, I73.9, I77.1, I79.0, I79.2, K55.1, K55.8, K55.9, Z95.8, Z95.9
Cerebrovascular disease	G45.x, G46.x, H34.0, I60.x–I69.x
Dementia	F00.x–F03.x, F05.1, G30.x, G31.1
Chronic pulmonary disease	I27.8, I27.9, J40.x–J47.x, J60.x–J67.x, J68.4, J70.1, J70.3
Rheumatic disease	M05.x, M06.x, M31.5, M32.x–M34.x, M35.1, M35.3, M36.0
Peptic ulcer disease	K25.x–K28.x
Mild liver disease	B18.x, K70.0–K70.3, K70.9, K71.3–K71.5, K71.7, K73.x, K74.x, K76.0, K76.2–K76.4, K76.8, K76.9, Z94.4
Diabetes without chronic complication	E10.0, E10.1, E10.6, E10.8, E10.9, E11.0, E11.1, E11.6, E11.8, E11.9, E12.0, E12.1, E12.6, E12.8, E12.9, E13.0, E13.1, E13.6, E13.8, E13.9, E14.0, E14.1, E14.6, E14.8, E14.9
Diabetes with chronic complication	E10.2–E10.5, E10.7, E11.2–E11.5, E11.7, E12.2–E12.5, E12.7, E13.2–E13.5, E13.7, E14.2–E14.5, E14.7
Hemiplegia or paraplegia	G04.1, G11.4, G80.1, G80.2, G81.x, G82.x, G83.0–G83.4, G83.9
Renal disease	I12.0, I13.1, N03.2–N03.7, N05.2–N05.7, N18.x, N19.x, N25.0, Z49.0–Z49.2, Z94.0, Z99.2
Any malignancy, including lymphoma and leukaemia, except malignant neoplasm of skin	C00.x–C26.x, C30.x–C34.x, C37.x–C41.x, C43.x, C45.x–C58.x, C60.x–C76.x, C81.x–C85.x, C88.x, C90.x–C97.x
Moderate or severe liver disease	I85.0, I85.9, I86.4, I98.2, K70.4, K71.1, K72.1, K72.9, K76.5, K76.6, K76.7
Metastatic solid tumour	C77.x–C80.x
AIDS/HIV	B20.x–B22.x, B24.x

All comorbidities are assigned a weight of 1 except hemiplegia/paraplegia, renal disease, and malignancies (weight=2); moderate/severe liver disease (weight=3); metastatic solid tumour and AIDS/HIV (weight=6). For diseases with two levels of severity (liver disease, diabetes and cancer), the less severe version is assigned weight=0 if the more severe version is also present in a patient. Reproduced from documentation for the 'comorbidity' R package (Gasparini, 2019)

Supplementary Table S2 | Descriptive characteristics at TP2 in 9814 men and women aged 48–92 by 10-year Charlson Comorbidity Index, 2009–2019

	Total	No admissions	CCI=0	CCI=1	CCI=2	CCI≥3
**Hospital duration 2009–2019, days**						
Mean ±SD	17.7 ±36.2	0.0 ±0.0	6.5 ±14.0	19.3 ±42.8	26.1 ±42.2	46.3 ±48.1
**Total hospital admissions 2009–2019**						
Mean ±SD	4.4 ±7.9	0.0 ±0.0	2.8 ±3.0	4.5 ±6.0	6.0 ±6.7	10.1 ±13.5
**Age at TP2, years**						
Mean ±SD	69.4 ±8.4	65.1 ±7.7	67.6 ±7.7	70.5 ±8.1	71.7 ±8.1	74.1 ±7.8
**Sex (n (%))**						
Men	4252	695 (16.3)	1294 (30.4)	631 (14.8)	558 (13.1)	1074 (25.3)
Women	5562	1166 (21.0)	1956 (35.2)	914 (16.4)	618 (11.1)	908 (16.3)
**Body mass index at TP2, kg/m²**						
Mean ±SD	26.9 ±4.3	26.2 ±4.3	26.5 ±4.0	27.3 ±4.4	27.3 ±4.5	27.7 ±4.5
**Cigarette smoking at TP2 (n (%))**						
Current	442	88 (19.9)	116 (26.2)	65 (14.7)	79 (17.9)	94 (21.3)
Former	4508	755 (16.7)	1375 (30.5)	741 (16.4)	565 (12.5)	1072 (23.8)
Never	4864	1018 (20.9)	1759 (36.2)	739 (15.2)	532 (10.9)	816 (16.8)
**Social class dichotomised (n (%))**						
Non-manual	6294	1205 (19.1)	2127 (33.8)	993 (15.8)	749 (11.9)	1220 (19.4)
Manual	3411	636 (18.6)	1087 (31.9)	528 (15.5)	424 (12.4)	736 (21.6)
**Level of education (n (%))**						
Higher level	7025	1460 (20.8)	2419 (34.4)	1074 (15.3)	791 (11.3)	1281 (18.2)
Lower level	2787	401 (14.4)	830 (29.8)	471 (16.9)	385 (13.8)	700 (25.1)
**Simple physical activity index at TP2 (n (%))**						
Inactive	3924	592 (15.1)	1072 (27.3)	687 (17.5)	545 (13.9)	1028 (26.2)
Moderately inactive	2682	555 (20.7)	940 (35.0)	404 (15.1)	311 (11.6)	472 (17.6)
Moderately active	1654	387 (23.4)	604 (36.5)	231 (14.0)	167 (10.1)	265 (16.0)
Active	1442	313 (21.7)	601 (41.7)	195 (13.5)	139 (9.6)	194 (13.5)
**Alcohol intake at TP2, units per week**						
Mean ±SD	5.7 ±8.2	6.3 ±8.0	5.8 ±7.9	5.5 ±8.3	5.3 ±7.8	5.5 ±9.1
**Plasma vitamin C at TP2, μmol/L**						
Mean ±SD	63.0 ±22.2	66.0 ±21.5	65.7 ±21.6	63.2 ±22.8	59.9 ±22.4	57.7 ±21.9
**Systolic blood pressure at TP2, mmHg**						
Mean ±SD	136.5 ±17.1	134.7 ±15.9	135.8 ±16.3	138.5 ±18.4	136.6 ±16.8	138.0 ±18.1
**Total cholesterol at TP2, mmol/L**						
Mean ±SD	5.4 ±1.1	5.6 ±1.1	5.5 ±1.1	5.3 ±1.1	5.2 ±1.2	5.0 ±1.2
**Prevalent heart attack at TP2 (n (%))**						
No reported heart attack at TP2	9455	1833 (19.4)	3211 (34.0)	1499 (15.9)	1116 (11.8)	1796 (19.0)
Self-reported heart attack at TP2	359	28 (7.8)	39 (10.9)	46 (12.8)	60 (16.7)	186 (51.8)
**Prevalent stroke at TP2 (n (%))**						
No reported stroke at TP2	9577	1843 (19.2)	3215 (33.6)	1510 (15.8)	1141 (11.9)	1868 (19.5)
Self-reported stroke at TP2	237	18 (7.6)	35 (14.8)	35 (14.8)	35 (14.8)	114 (48.1)
**Prevalent cancer at TP2 (n (%))**						
No reported cancer at TP2	8888	1744 (19.6)	2987 (33.6)	1398 (15.7)	1052 (11.8)	1707 (19.2)
Self-reported cancer at TP2	926	117 (12.6)	263 (28.4)	147 (15.9)	124 (13.4)	275 (29.7)
**Prevalent diabetes at TP2 (n (%))**						
No reported diabetes at TP2	9477	1834 (19.4)	3238 (34.2)	1477 (15.6)	1124 (11.9)	1804 (19.0)
Self-reported diabetes at TP2	337	27 (8.0)	12 (3.6)	68 (20.2)	52 (15.4)	178 (52.8)

Supplementary Table S3 | Multivariable logistic regression of risk factors for Charlson 5-year and 10-year hospital admissions with multimorbidity at TP2 in 9814 men and women

	Charlson 5-year multimorbidity †, 2009–2014 OR (95% CI)	p value	Charlson 10-year multimorbidity †, 2009–2019 OR (95% CI)	p value
**Model 1**				
Male sex	1.67 (1.46–1.91)	< 0.001	1.72 (1.55–1.91)	< 0.001
Age per 10 years	2.35 (2.16–2.56)	< 0.001	2.36 (2.21–2.53)	< 0.001
Manual social class at baseline	1.02 (0.88–1.18)	0.774	1.11 (0.99–1.24)	0.072
Lower education level at baseline	1.12 (0.96–1.30)	0.154	1.26 (1.12–1.42)	< 0.001
**Model 2**				
Male sex	1.60 (1.39–1.84)	< 0.001	1.65 (1.48–1.84)	< 0.001
Age per 10 years	2.19 (2.00–2.39)	< 0.001	2.22 (2.07–2.38)	< 0.001
Manual social class at baseline	1.01 (0.87–1.18)	0.850	1.11 (0.99–1.24)	0.082
Lower education level at baseline	1.10 (0.94–1.29)	0.215	1.25 (1.11–1.41)	< 0.001
Prevalent CVD	2.25 (1.78–2.81)	< 0.001	2.60 (2.13–3.18)	< 0.001
Prevalent cancer	1.83 (1.50–2.22)	< 0.001	1.61 (1.37–1.90)	< 0.001
Prevalent diabetes	3.96 (3.08–5.08)	< 0.001	3.91 (3.09–4.96)	< 0.001
**Model 3**				
Male sex	1.44 (1.24–1.67)	< 0.001	1.52 (1.35–1.71)	< 0.001
Age per 10 years	2.14 (1.95–2.35)	< 0.001	2.23 (2.07–2.39)	< 0.001
Manual social class at baseline	0.97 (0.83–1.13)	0.692	1.07 (0.95–1.20)	0.287
Lower education level at baseline	1.04 (0.89–1.22)	0.598	1.20 (1.06–1.35)	0.004
Current smoker	1.44 (1.02–1.99)	0.032	1.45 (1.11–1.86)	0.005
BMI>30 kg/m²	1.38 (1.17–1.63)	< 0.001	1.54 (1.35–1.75)	< 0.001
Alcohol intake, units per week	1.00 (0.99–1.01)	0.611	1.00 (0.99–1.01)	0.664
Physically inactive	1.30 (1.12–1.50)	< 0.001	1.13 (1.01–1.26)	0.034
Plasma vitamin C per SD	0.80 (0.74–0.87)	< 0.001	0.83 (0.79–0.88)	< 0.001
Prevalent CVD	2.11 (1.67–2.64)	< 0.001	2.46 (2.01–3.02)	< 0.001
Prevalent cancer	1.81 (1.48–2.20)	< 0.001	1.61 (1.36–1.89)	< 0.001
Prevalent diabetes	3.55 (2.75–4.56)	< 0.001	3.47 (2.74–4.41)	< 0.001
**Model 4**				
Male sex	1.35 (1.15–1.58)	< 0.001	1.41 (1.25–1.60)	< 0.001
Age per 10 years	2.11 (1.92–2.32)	< 0.001	2.20 (2.04–2.37)	< 0.001
Manual social class at baseline	0.97 (0.83–1.13)	0.692	1.07 (0.95–1.20)	0.287
Lower education level at baseline	1.04 (0.89–1.22)	0.609	1.20 (1.06–1.35)	0.004
Current smoker	1.43 (1.02–1.98)	0.034	1.44 (1.10–1.85)	0.006
BMI>30 kg/m²	1.37 (1.16–1.62)	< 0.001	1.53 (1.34–1.74)	< 0.001
Alcohol intake, units per week	1.00 (0.99–1.01)	0.449	1.00 (1.00–1.01)	0.416
Physically inactive	1.29 (1.12–1.50)	< 0.001	1.13 (1.01–1.26)	0.038
Plasma vitamin C per SD	0.81 (0.75–0.87)	< 0.001	0.84 (0.79–0.89)	< 0.001
Systolic blood pressure per SD	0.99 (0.92–1.07)	0.854	0.99 (0.94–1.05)	0.769
Total cholesterol per SD	0.91 (0.84–0.98)	0.014	0.89 (0.84–0.95)	< 0.001
Prevalent CVD	2.02 (1.60–2.54)	< 0.001	2.34 (1.91–2.87)	< 0.001
Prevalent cancer	1.81 (1.48–2.20)	< 0.001	1.60 (1.36–1.89)	< 0.001
Prevalent diabetes	3.28 (2.52–4.24)	< 0.001	3.16 (2.48–4.03)	< 0.001

† Charlson Comorbidity Index ≥3 vs Charlson Comorbidity Index ≤2 or no hospital admission.

Supplementary Table S4 | Multivariable logistic regression of risk factors excluding participants with prevalent CVD, cancer or diabetes at TP2 for 5-year and 10-year hospital admissions with multimorbidity at TP2 in 8185 men and women

	5-year follow-up period †, 2009–2014 OR (95% CI)	p value	10-year follow-up period †, 2009–2019 OR (95% CI)	p value
**Model 1**				
Male sex	1.44 (1.22–1.70)	< 0.001	1.55 (1.37–1.75)	< 0.001
Age per 10 years	2.36 (2.13–2.62)	< 0.001	2.39 (2.21–2.58)	< 0.001
Manual social class at baseline	0.99 (0.82–1.18)	0.888	1.11 (0.97–1.27)	0.122
Lower education level at baseline	1.12 (0.93–1.36)	0.220	1.26 (1.09–1.44)	0.001
**Model 2**				
Male sex	1.25 (1.05–1.50)	0.015	1.39 (1.22–1.59)	< 0.001
Age per 10 years	2.36 (2.12–2.63)	< 0.001	2.43 (2.24–2.64)	< 0.001
Manual social class at baseline	0.93 (0.78–1.12)	0.471	1.06 (0.93–1.22)	0.373
Lower education level at baseline	1.06 (0.88–1.29)	0.518	1.21 (1.05–1.39)	0.008
Current smoker	1.81 (1.23–2.59)	0.002	1.66 (1.24–2.20)	< 0.001
BMI>30 kg/m²	1.42 (1.16–1.73)	< 0.001	1.60 (1.38–1.86)	< 0.001
Alcohol intake, units per week	1.01 (1.00–1.02)	0.238	1.01 (1.00–1.01)	0.109
Physically inactive	1.18 (0.99–1.41)	0.061	1.10 (0.97–1.26)	0.138
Plasma vitamin C per SD	0.79 (0.72–0.86)	< 0.001	0.83 (0.77–0.88)	< 0.001
**Model 3**				
Male sex	1.16 (0.96–1.40)	0.135	1.29 (1.12–1.49)	< 0.001
Age per 10 years	2.29 (2.05–2.57)	< 0.001	2.39 (2.20–2.60)	< 0.001
Manual social class at baseline	0.93 (0.77–1.12)	0.446	1.06 (0.93–1.21)	0.385
Lower education level at baseline	1.06 (0.88–1.28)	0.534	1.21 (1.05–1.39)	0.009
Current smoker	1.81 (1.23–2.59)	0.002	1.64 (1.23–2.18)	< 0.001
BMI>30 kg/m²	1.39 (1.14–1.70)	0.001	1.59 (1.36–1.84)	< 0.001
Alcohol intake, units per week	1.01 (1.00–1.02)	0.163	1.01 (1.00–1.02)	0.054
Physically inactive	1.18 (0.99–1.40)	0.071	1.10 (0.96–1.25)	0.161
Plasma vitamin C per SD	0.79 (0.72–0.87)	< 0.001	0.83 (0.77–0.89)	< 0.001
Systolic blood pressure per SD	1.03 (0.95–1.12)	0.466	0.99 (0.93–1.06)	0.866
Total cholesterol per SD	0.88 (0.80–0.96)	0.005	0.89 (0.83–0.95)	< 0.001

† Charlson Comorbidity Index ≥3 vs Charlson ≤2 or no hospital admission.
