# Supplementary material for: Sociodemographic and lifestyle predictors of incident hospital admissions with multimorbidity in a general population, 1999–2019: the EPIC-Norfolk cohort
Source: BMJ Open. 2020 Sep 22;10(9):e042115. doi: 10.1136/bmjopen-2020-042115 (PMC7509968; doi:10.1136/bmjopen-2020-042115)
Supplement: Author's manuscript [file bmjopen-2020-042115.draft_revisions.pdf]

BMJ Open is committed to open peer review. As part of this commitment we make the peer review history of every article we publish publicly available.

When an article is published we post the peer reviewers' comments and the authors' responses online. We also post the versions of the paper that were used during peer review. These are the versions that the peer review comments apply to.

The versions of the paper that follow are the versions that were submitted during the peer review process. They are not the versions of record or the final published versions. They should not be cited or distributed as the published version of this manuscript.

BMJ Open is an open access journal and the full, final, typeset and author-corrected version of record of the manuscript is available on our site with no access controls, subscription charges or pay-per-view fees (http://bmjopen.bmj.com).

If you have any questions on BMJ Open's open peer review process please email info.bmjopen@bmj.com

BMJ Open

Sociodemographic and lifestyle predictors of incident hospital admissions with multimorbidity in a general population 1999–2019: the EPIC-Norfolk cohort

Journal:	*BMJ Open*
Manuscript ID	bmjopen-2020-042115
Article Type:	Original research
Date Submitted by the Author:	25-Jun-2020
Complete List of Authors:	Luben, Robert; University of Cambridge School of Clinical Medicine, Department of Public Health and Primary Care<br>Hayat, Shabina; University of Cambridge School of Clinical Medicine, Department of Public Health and Primary Care<br>Wareham, Nicholas; University of Cambridge School of Clinical Medicine, MRC Epidemiology Unit<br>Pharoah, Paul; University of Cambridge School of Clinical Medicine, Department of Public Health and Primary Care<br>Khaw, Kay-Tee; University of Cambridge School of Clinical Medicine, MRC Epidemiology Unit
Keywords:	EPIDEMIOLOGY, Health policy < HEALTH SERVICES ADMINISTRATION & MANAGEMENT, SOCIAL MEDICINE

SCHOLARONE™
Manuscripts

I, the Submitting Author has the right to grant and does grant on behalf of all authors of the Work (as defined in the below author licence), an exclusive licence and/or a non-exclusive licence for contributions from authors who are: i) UK Crown employees; ii) where BMJ has agreed a CC-BY licence shall apply, and/or iii) in accordance with the terms applicable for US Federal Government officers or employees acting as part of their official duties; on a worldwide, perpetual, irrevocable, royalty-free basis to BMJ Publishing Group Ltd ("BMJ") its licensees and where the relevant Journal is co-owned by BMJ to the co-owners of the Journal, to publish the Work in this journal and any other BMJ products and to exploit all rights, as set out in our licence.

The Submitting Author accepts and understands that any supply made under these terms is made by BMJ to the Submitting Author unless you are acting as an employee on behalf of your employer or a postgraduate student of an affiliated institution which is paying any applicable article publishing charge ("APC") for Open Access articles. Where the Submitting Author wishes to make the Work available on an Open Access basis (and intends to pay the relevant APC), the terms of reuse of such Open Access shall be governed by a Creative Commons licence – details of these licences and which Creative Commons licence will apply to this Work are set out in our licence referred to above.

Other than as permitted in any relevant BMJ Author's Self Archiving Policies, I confirm this Work has not been accepted for publication elsewhere, is not being considered for publication elsewhere and does not duplicate material already published. I confirm all authors consent to publication of this Work and authorise the granting of this licence.

For peer review only

Sociodemographic and lifestyle predictors of incident hospital admissions with multimorbidity in a general population 1999–2019: the EPIC-Norfolk cohort

Robert Luben[1], Shabina Hayat[1], Nicholas Wareham[2], Paul Pharoah[1], Kay-Tee Khaw[1]

1 Department of Public Health and Primary Care, Institute of Public Health, University of Cambridge, Cambridge, United Kingdom
2 MRC Epidemiology Unit, University of Cambridge School of Clinical Medicine, Cambridge United Kingdom
Correspondence to: Robert Luben, Department of Public Health and Primary Care, Strangeways Research Laboratory, Worts Causeway, Cambridge, UK. Email: robert.luben@phpc.cam.ac.uk Telephone: +44 (0)1223 748691

June 22, 2020

For peer review only

Abstract

Background

The ageing population and prevalence of long-term disorders with multimorbidity is a major health challenge worldwide. The associations between comorbid conditions and mortality risk are well established; however, few prospective community-based studies have reported on prior risk factors for incident hospitalisations with multimorbidity. We aimed to explore the independent associations for a range of demographic, lifestyle and physiological determinants and the likelihood of subsequent hospital incident multimorbidity.

Methods

We examined incident hospital admissions with multimorbidity in 25014 men and women aged 40–79 in a British prospective population-based study recruited 1993–1997 and followed-up until 2019. The determinants of incident multimorbidity, defined as Charlson Comorbidity Index ≥3, were investigated using multivariable models for the 10-year period 1999–2009 and repeated with independent measurements in a second 10-year period 2009–2019.

Results

Between 1999–2009 18179 participants (73% of the population) had a hospital admission. Baseline 5-year and 10-year incident multimorbidity were observed in 13% and 21% of participants respectively. Age per 10-year increase OR 2.19 (95%CI 2.06–2.33) and male sex OR 1.32 (95%CI 1.19–1.47) predicted incident multimorbidity over 10 years. In the subset free of the most serious diseases at baseline, current smoking OR 1.74 (95%CI 1.52–2.00), BMI >30 kg/m^2 OR 1.40 (95%CI 1.24–1.58) and physical inactivity OR 1.14 (95%CI 1.04–1.26) were positively associated and plasma vitamin C (a biomarker of plant food intake) per SD increase OR 0.85 (95%CI 0.81–0.89) inversely associated with incident 10-year multimorbidity after multivariable adjustment for age, sex, social class, education, alcohol consumption, systolic blood pressure and cholesterol. Results were similar when re-examined for a further time period 2009–2019.

Conclusion

Age, male sex and potentially modifiable lifestyle behaviours including smoking, body mass index, physical inactivity and low fruit and vegetable intake were associated with increased risk of future incident hospital admissions with multimorbidity.

For peer review only - http://bmjopen.bmj.com/site/about/guidelines.xhtml

Article summary

Strengths and limitations of this study

- The majority of patients in secondary care are elderly and have multiple chronic conditions.
- Multimorbidity predicts future increased mortality but most studies are conducted in individuals who access health care.
- Cross-sectional studies have reported associations with lifestyle factors but there are few prospective population-based studies of predictors of future multimorbidity.
- In this population-based prospective study, followed-up over 20 years, we examined the likelihood of hospitalisation with multimorbidity by demographic characteristics including age and sex.
- We examined the demographic, lifestyle and physiological risk factors that predict incident multimorbidity in 5 and 10-year periods spanning 20 years and identified potentially modifiable lifestyle factors such as smoking, obesity, physical inactivity and low plant food intake.

Acknowledgements: *The authors would like to thank the participants, general practitioners, and staff of EPIC-Norfolk.*

Contributors: *KTK,NW SH and RL were involved in the conception and design of the study. RL drafted the manuscript, with support from KTK and PP. SH contributed to data interpretation. RL was responsible for external data linkage. SH and RL contributed to data collection and acquisition. All authors read and critically revised the manuscript and approved the final manuscript. RL is the guarantor. The corresponding author attests that all listed authors meet authorship criteria and that no others meeting the criteria have been omitted.*

Funding: *The design and conduct of the EPIC-Norfolk study and collection and management of the data was supported by programme grants from the Medical Research Council UK (G9502233, G0401527) and Cancer Research UK (C864/A8257, C864/A2883).*

Competing interests: *All authors have completed the ICMJE uniform disclosure form at www.icmje.org/coi_disclosure.pdf and report: RL,SH,KTK and NW report grants from MRC and CRUK during the conduct of the study; The sponsors had no role in any of the following: study design, data collection, data analysis, interpretation of data, writing of the article, decision to submit it for publication. All authors are independent of funders and sponsors and had access to all the data. No conflicts of interest were declared by any author (apart from the two grants) and they have no other relationships or activities that could appear to have influenced the submitted work.*

Ethical approval: *The work was approval by the East Norfolk and Waveney NHS research governance committee (2005EC07L) and the Norfolk research ethics committee (05/Q0101/191). All participants gave informed signed consent for study participation including access to medical records.*

Data sharing: *The authors will make the dataset available under a Data Transfer Agreement to any bona fide researcher who wishes to obtain the dataset in order to undertake a replication analysis. Although the dataset is anonymised, the breadth of the data included and the multiplicity of variables that are included in this analysis file as primary variables or confounding factors, means that provision of the dataset to other researchers without a Data Transfer Agreement would constitute a risk. Requests for data sharing/access should be submitted to the EPIC Management Committee (epic-norfolk@mrc-epid.cam.ac.uk)*

Transparency: *The lead author (the manuscript's guarantor) affirms that the manuscript is an honest, accurate, and transparent account of the study being reported; that no important aspects of the study have been omitted; and that any discrepancies from the study as planned (and, if relevant, registered) have been explained.*

Open access: *This is an Open Access article distributed in accordance with the terms of the Creative Commons Attribution (CC BY 4.0) license, which permits others to distribute, remix, adapt and build upon this work, for commercial use, provided the original work is properly cited. See: http://creativecommons.org/licenses/by/4.0/*

Patient and public involvement: *The EPIC-Norfolk Study have an active Participants Advisory Panel which meets quarterly to advise on research protocols, suggest ideas and provide feedback on the research including proposed new studies and collaborations. All participants of the EPIC-Norfolk study are informed about the study through regular newsletters as well as public meetings. Information is also disseminated through local community talks in the Norfolk area and science festivals.*

Exclusive licence: *The Corresponding Author has the right to grant on behalf of all authors and does grant on behalf of all authors, a worldwide licence to the Publishers and its licensees in perpetuity, in all forms, formats and media (whether known*

For peer review only - http://bmjopen.bmj.com/site/about/guidelines.xhtml

now or created in the future), to i) publish, reproduce, distribute, display and store the Contribution, ii) translate the Contribution into other languages, create adaptations, reprints, include within collections and create summaries, extracts and/or, abstracts of the Contribution, iii) create any other derivative work(s) based on the Contribution, iv) to exploit all subsidiary rights in the Contribution, v) the inclusion of electronic links from the Contribution to third party material where-ever it may be located; and, vi) licence any third party to do any or all of the above.

For peer review only

For peer review only - http://bmjopen.bmj.com/site/about/guidelines.xhtml

Introduction

The Academy of Medical Sciences 2018 report highlighted multimorbidity as a global priority for research. Patients with multimorbidity experience reduced wellbeing and quality of life and account for a disproportionately high share of healthcare workload and costs. Management of the rising prevalence of long-term disorders is the main challenge facing health-care systems worldwide. [1–3]

Multimorbidity is commonly defined as the presence of multiple diseases or conditions with a cut-off of two or more conditions [4], however there is no agreed definition or classification system, which makes the existing evidence base difficult to interpret. [1] The term comorbidity predates multimorbidity and was used to predict the effect of additional diseases for those with an index disease of interest. [5–7] The Charlson Comorbidity Index (CCI) [8] was originally created to predict mortality in hospital patients after one year and is defined using a set of 17 chronic diseases, weighted according to the risk of death. The index has been widely used with several authors suggesting extensions or modifications to the original definition [9–14] and it remains a common standard to which other systems are often compared. [15]

The associations between comorbid conditions and mortality are well established. [16–20] However, few studies have examined the determinants of incident multimorbidity rather than its consequences [21–26] since most lack detailed demographic, socioeconomic and physiological measurements in population-based men and women prior to the onset of multimorbid disease with subsequent follow-up. Retrospective hospital-based studies examining multimorbidity lack community-based denominators while general practice-based studies are often cross-sectional or examine mortality in already multimorbid patients. Few studies examine factors that predict the likelihood of multimorbidity rather than factors that predict risk of individual component conditions. The large majority of studies conducted to date are cross-sectional, with few prospective community-based studies able to examine incident multimorbidity from subsequent hospitalisation. [1,24,25] In this study, we examine the independent associations for a range of demographic, lifestyle and physiological determinants and the likelihood of subsequent hospital incident multimorbidity. We use the CCI over 5-year and 10-year time periods and re-examine these associations independently in a subset 12 years after baseline since health care policy and the criteria used for admission may have changed over time. We have previously reported on risk factors for hospitalisation [27–29] but here we explore in more detail hospital admissions with multimorbidity, a measure of both health service and individual burden.

Methods

We used data from the European Prospective Investigation into Cancer in Norfolk cohort (EPIC-

For peer review only - http://bmjopen.bmj.com/site/about/guidelines.xhtml

Norfolk) . [30,31] 25639 men and women aged 40–79 were recruited from general practices in Norfolk, completed a lifestyle questionnaire and attended a baseline health check from 1993 to 1997. Participants were reapproached approximately 12 years later, aged 48–92 with 9814 completing a second questionnaire and 8049 attending a health check at time-point two (TP2). Figure 1 shows a flow diagram of participant numbers at various stages. The cohort was followed-up until 2019 with annual record linkage to hospital episode data. Since linkage was to national databases and migration of cohort participants was rare, there was almost no loss to follow-up.

The Charlson Comorbidity Index (CCI) is defined using a set of chronic diseases, each having an associated weight (1, 2, 3 or 6) related to the risk of death. The conditions are myocardial infarction, congestive heart failure, peripheral vascular disease, cerebrovascular disease, dementia, chronic pulmonary disease, rheumatoid disease, peptic ulcer disease, liver disease, diabetes, hemiplegia or paraplegia, renal disease, cancer and AIDS/HIV. Two levels of severity are defined for liver disease, diabetes and cancer (details are shown in supplementary table S4). All comorbidities are assigned a weight of 1 except hemiplegia/paraplegia, renal disease, and malignancies (weight=2); moderate/severe liver disease (weight=3); metastatic solid tumour and AIDS/HIV (weight=6). For diseases with two levels of severity (liver disease, diabetes and cancer), the less severe version is assigned weight=0 if the more severe version is also present in a patient. The CCI diseases were assigned diagnosis codes using the International Classification of Diseases (ICD-10) which was used to link the CCI to hospital episode statistics (HES) records and to cohort participants. The weighted individual disease scores were totalled to create an overall score with a maximum value of 29. [8,12] CCI was measured for various outcome periods restricted to all hospital events within the given time period: at baseline, 5-year (1999–2004) and 10-year (1999–2009) CCI and at TP2, 5-year (2009–2014) and 10-year (2009–2019) CCI. Multiple admissions including the same CCI category were only counted once.

Participants attending the baseline and TP2 health examinations had their height to the nearest 0.1 cm measured using a stadiometer (Chasemores, UK) and their weight to the nearest 100g measured in light clothing without shoes (Salter, West Bromwich, UK). Body mass index (BMI) was calculated using measured weight in kilograms divided by the square of measured height in square metres. Trained nurses obtained non-fasting blood samples by venepuncture into plain and citrate bottles. Bloods were assayed at the Department of Clinical Biochemistry, University of Cambridge, UK. Serum concentrations of total cholesterol were measured with the RA 1000 Technicon analyser (Bayer Diagnostics, Basingstoke). Plasma was stabilised in a standardised volume of metaphosphoric acid stored at −70 °C and vitamin C concentrations measured using a fluorometric assay within one week. [32] Systolic blood pressure was measured using an Accutorr Sphygmomanometer (Datascope Medical, Huntington, United Kingdom). Participants sat for three minutes before two measurements

For peer review only - http://bmjopen.bmj.com/site/about/guidelines.xhtml

were taken with the arm horizontal and held at mid-sternum level. Systolic blood pressure was defined as the average of the two measurements.

At baseline and again at TP2, participants completed a lifestyle questionnaire. Two yes/no questions were used to derive smoking status: "Have you ever smoked as much as one cigarette a day for as long as a year?" and, where a positive response was given, "Do you smoke cigarettes now?" Participants also completed questions about their employment and that of their partner with details of both current and past employment recorded. Occupational social class was defined according to the Registrar General's classification. [33,34] A list of common UK qualifications was used to establish educational attainment and participants were asked to mark all relevant qualifications. These were then categorised using the highest qualification attained. Participants were asked about their occupational and leisure physical activity. A combined score was created combining leisure and occupational elements and divided into four ordered categories with those who did not complete the question placed in the inactive category. The score was validated against energy expenditure measured by free-living heart rate monitoring with individual calibration. [35,36] Participants were asked "Are you a non-drinker/teetotaller now?" and "At present, about how many alcoholic drinks do you have each week" for various types of alcohol. Current units were calculated from the questionnaire responses with one unit equal to a half pint of beer, one glass of wine or fortified wine or a single measure of spirits. Prevalent disease was established from the question "Has the doctor ever told you that you have any of the following?" followed by a list of common conditions including "Heart attack (myocardial infarction)", "Stroke", "Cancer" and "Diabetes".

Statistical methods

Associations were examined both including and excluding chronic disease at baseline and repeated with independent measurements at TP2 in a subset of participants using a second baseline 12 years approximately after the first. The baseline analysis excludes 625 men and women who died before 1999 while at TP2 a further 126 participants who died prior to 2009 were excluded. Dichotomous variables were created for the social class (manual and non-manual), educational attainment (high and low) at baseline and body mass index (>30 and ≤30 kg/m^2) and usual physical activity (active and inactive) at both baseline and TP2. For social class, professional, managerial and technical and non-manual skilled occupations were classed as non-manual while manual skilled, partly skilled and unskilled were classed as manual. For educational attainment, those with qualifications at secondary level or above where classed as high and those with no qualification as low. Hospital outcomes were categorised into five groups, "No hospital admissions", CCI=0, CCI=1, CCI=2 and CCI ≥3. Multivariable logistic regression was used for all models comparing CCI ≤2 with CCI ≥3. A sensitivity analysis, using identical models to those in table 4 for the period 1999–2009, but excluding 80 participants defined as multimorbid having only one condition with a CCI weighting ≥3, gave virtually identical results to table

For peer review only - http://bmjopen.bmj.com/site/about/guidelines.xhtml

4 (results not shown).

The numbers of individuals with missing values for covariables at baseline were: 53 BMI, 218 smoking status, 545 social class, 18 education level. The physical activity score has no missing values since those with missing data were classified as being inactive. Multiple imputation was used to estimate missing values at TP2 most apparent when participants completed questionnaires but did not attend a health examination n=1891. Predictive mean matching with 5 multiple imputations and 50 iterations was used with baseline and TP2 variables. All analyses were performed using the R statistical language (R Foundation for Statistical Computing, Vienna, Austria V3.5.3 with packages knitr, Gmisc, ggplot2, tidyverse, intubate, mice.) CCIs were calculated using the R package "comorbidity". [37]

Results

Table 1 shows future 5-year and 10-year multimorbidity hospital admission rates from baseline for n=25014 and from time-point two (TP2) for n=9814 according to demographic characteristics in the study population. Between 1999–2009 18179 participants (73% of the population) had a hospital admission. Baseline 5-year and 10-year incident multimorbidity were observed in 13% and 21% of participants respectively. Figure 2 shows 10-year multimorbidity rates (CCI ≥2) by age group and sex. More men had CCI ≥3 than women and those aged >75 years had the highest proportion of admissions with multimorbid conditions with 30.6% at 5 years and 36.4% at 10 years. Multimorbidity rates at TP2 were similar to baseline with 5-year and 10-year incident CCI ≥3 observed in 20% and 28% of participants respectively and similar rates in those >75 years.

Table 1 | Charlson Comorbidity Index hospitalisation rates by age-group and sex in men and women aged 40–79, 1999–2019

	Total	No admissions	Charlson 0	Charlson 1	Charlson 2	Charlson ≥3
**Baseline 5-year multimorbidity, 1999–2004 (n(%))**						
Men	11228	5457 (48.6)	3340 (29.7)	988 (8.8)	662 (5.9)	781 (7.0)
Women	13786	7153 (51.9)	4398 (31.9)	953 (6.9)	643 (4.7)	639 (4.6)
≤55 years	9567	6009 (62.8)	2720 (28.4)	411 (4.3)	236 (2.5)	191 (2.0)
(55–65] years	7805	3940 (50.5)	2479 (31.8)	583 (7.5)	408 (5.2)	395 (5.1)
(65–75] years	6933	2489 (35.9)	2322 (33.5)	830 (12.0)	561 (8.1)	731 (10.5)
>75 years	709	172 (24.3)	217 (30.6)	117 (16.5)	100 (14.1)	103 (14.5)
**Baseline 10-year multimorbidity, 1999–2009 (n(%))**						
Men	11228	2928 (26.1)	4151 (37.0)	1434 (12.8)	1056 (9.4)	1659 (14.8)
Women	13786	3907 (28.3)	5767 (41.8)	1601 (11.6)	1137 (8.2)	1374 (10.0)
≤55 years	9567	3720 (38.9)	4201 (43.9)	746 (7.8)	476 (5.0)	424 (4.4)
(55–65] years	7805	1973 (25.3)	3259 (41.8)	994 (12.7)	711 (9.1)	868 (11.1)
(65–75] years	6933	1059 (15.3)	2294 (33.1)	1168 (16.8)	875 (12.6)	1537 (22.2)
>75 years	709	83 (11.7)	164 (23.1)	127 (17.9)	131 (18.5)	204 (28.8)
**Time-point two 5-year multimorbidity, 2009–2014 (n(%))**						
Men	4252	1428 (33.6)	1355 (31.9)	522 (12.3)	389 (9.1)	558 (13.1)
Women	5562	2234 (40.2)	1793 (32.2)	686 (12.3)	403 (7.2)	446 (8.0)
≤55 years	342	215 (62.9)	92 (26.9)	19 (5.6)	10 (2.9)	6 (1.8)
(55–65] years	3090	1540 (49.8)	1006 (32.6)	277 (9.0)	143 (4.6)	124 (4.0)
(65–75] years	3695	1303 (35.3)	1301 (35.2)	464 (12.6)	286 (7.7)	341 (9.2)
>75 years	2687	604 (22.5)	749 (27.9)	448 (16.7)	353 (13.1)	533 (19.8)
**Time-point two 10-year multimorbidity, 2009–2019 (n(%))**						

For peer review only - http://bmjopen.bmj.com/site/about/guidelines.xhtml

For peer review only

	Total	No admissions	Charlson 0	Charlson 1	Charlson 2	Charlson ≥3
Men	4252	695 (16.3)	1294 (30.4)	631 (14.8)	558 (13.1)	1074 (25.3)
Women	5562	1166 (21.0)	1956 (35.2)	914 (16.4)	618 (11.1)	908 (16.3)
≤55 years	342	154 (45.0)	122 (35.7)	37 (10.8)	14 (4.1)	15 (4.4)
(55–65] years	3090	905 (29.3)	1241 (40.2)	407 (13.2)	267 (8.6)	270 (8.7)
(65–75] years	3695	589 (15.9)	1309 (35.4)	611 (16.5)	473 (12.8)	713 (19.3)
>75 years	2687	213 (7.9)	578 (21.5)	490 (18.2)	422 (15.7)	984 (36.6)

Descriptive characteristics of the cohort according to 10-year CCI are shown in table 2. Participants with higher number of admissions and longer duration of hospital stay had higher CCI, with mean duration 58 days and 13 admissions for participants with CCI ≥3 during the 10-year period. Participants with multimorbidity admissions were more likely at baseline examination to be current smokers, less physically active, have higher body mass index (BMI) and have lower plasma vitamin C (a proxy for a diet rich in fruit and vegetables) and report various prevalent conditions.

Table 2 | Descriptive characteristics at baseline in 25014 men and women aged 40–79 by 10-year Charlson Comorbidity Index, 1999–2009

	Total	No admissions	Charlson 0	Charlson 1	Charlson 2	Charlson ≥3
**Hospital duration 1999–2009, days**						
Mean ±SD	16.3 ±46.5	0.0 ±0.0	9.1 ±28.3	24.9 ±71.5	30.4 ±43.0	57.8 ±77.5
**Total hospital admissions 1999–2009**						
Mean ±SD	3.8 ±16.2	0.0 ±0.0	2.8 ±3.1	4.5 ±6.0	6.4 ±8.3	13.4 ±43.9
**Age, years**						
Mean ±SD	59.0 ±9.3	55.4 ±8.6	57.9 ±8.8	62.0 ±8.8	62.9 ±8.8	65.0 ±8.0
**Body mass index, kg/m²**						
Mean ±SD	26.4 ±3.9	25.9 ±3.7	26.2 ±3.8	26.8 ±4.1	26.8 ±4.3	27.3 ±4.2
**Cigarette smoking (n (%))**						
Current	2904	751 (25.9)	1008 (34.7)	410 (14.1)	291 (10.0)	444 (15.3)
Former	10423	2558 (24.5)	4007 (38.4)	1352 (13.0)	979 (9.4)	1527 (14.7)
Never	11469	3476 (30.3)	4821 (42.0)	1245 (10.9)	903 (7.9)	1024 (8.9)
**Social class dichotomised (n (%))**						
Non-manual	14717	4400 (29.9)	5707 (38.8)	1733 (11.8)	1256 (8.5)	1621 (11.0)
Manual	9752	2304 (23.6)	4029 (41.3)	1214 (12.4)	886 (9.1)	1319 (13.5)
**Level of education (n (%))**						
Higher level	15866	4922 (31.0)	6333 (39.9)	1724 (10.9)	1277 (8.0)	1610 (10.1)
Lower level	9130	1910 (20.9)	3576 (39.2)	1310 (14.3)	916 (10.0)	1418 (15.5)
**Simple physical activity index (n (%))**						
Inactive	7559	1681 (22.2)	2666 (35.3)	1116 (14.8)	788 (10.4)	1308 (17.3)
Moderately inactive	7187	2084 (29.0)	2904 (40.4)	819 (11.4)	610 (8.5)	770 (10.7)
Moderately active	5688	1708 (30.0)	2353 (41.4)	608 (10.7)	470 (8.3)	549 (9.7)
Active	4580	1362 (29.7)	1995 (43.6)	492 (10.7)	325 (7.1)	406 (8.9)
**Alcohol intake, units per week**						
Mean ±SD	7.1 ±9.5	7.7 ±9.6	6.9 ±9.1	6.9 ±9.5	6.7 ±9.8	6.8 ±10.3
**Plasma vitamin C, µmol/L**						
Mean ±SD	53.5 ±20.3	55.3 ±19.8	55.4 ±19.9	50.5 ±20.3	51.3 ±20.9	47.6 ±20.6
**Systolic blood pressure, mmHg**						
Mean ±SD	135.3 ±18.3	132.4 ±17.4	133.5 ±17.5	138.7 ±18.7	138.6 ±19.2	142.2 ±19.3
**Total cholesterol, mmol/L**						
Mean ±SD	6.2 ±1.2	6.1 ±1.1	6.1 ±1.1	6.3 ±1.2	6.2 ±1.2	6.3 ±1.2
**Prevalent heart attack (n (%))**						
No reported heart attack	24253	6745 (27.8)	9764 (40.3)	2886 (11.9)	2097 (8.6)	2761 (11.4)
Self-reported heart attack	728	85 (11.7)	143 (19.6)	146 (20.1)	94 (12.9)	260 (35.7)
**Prevalent stroke (n (%))**						
No reported stroke	24660	6786 (27.5)	9821 (39.8)	2975 (12.1)	2151 (8.7)	2927 (11.9)
Self-reported stroke	329	45 (13.7)	87 (26.4)	57 (17.3)	41 (12.5)	99 (30.1)
**Prevalent cancer (n (%))**						
No reported cancer	23688	6595 (27.8)	9449 (39.9)	2878 (12.1)	2031 (8.6)	2735 (11.5)

For peer review only

For peer review only - http://bmjopen.bmj.com/site/about/guidelines.xhtml

	Total	No admissions	Charlson 0	Charlson 1	Charlson 2	Charlson ≥3
Self-reported cancer	1301	237 (18.2)	459 (35.3)	155 (11.9)	162 (12.5)	288 (22.1)
**Prevalent diabetes (n (%))**						
No reported diabetes	24442	6760 (27.7)	9844 (40.3)	2941 (12.0)	2111 (8.6)	2786 (11.4)
Self-reported diabetes	541	71 (13.1)	61 (11.3)	90 (16.6)	81 (15.0)	238 (44.0)

In table 3, odds ratios are shown for 5-year and 10-year incident multimorbidity, defined as those with CCI ≥3, compared with CCI ≤1 or no hospital admission, adjusted age, sex, occupational social class and educational attainment in model 1. Model 2 additionally adjusts for prevalent diseases, cardiovascular disease (CVD), cancer and diabetes; model 3 adds lifestyle factors, current smoking, alcohol units per week, usual physical activity as well as BMI >30 kg/m^2 and plasma vitamin C; model 4 adds systolic blood pressure and cholesterol. Age, sex and prevalent diseases were strongly associated with multimorbidity admissions in all models. The fully adjusted association of 10-year incident multimorbidity with age per 10 years increase was OR 2.19 (95% CI 2.06–2.33), for sex OR 1.32 (95% CI 1.19–1.47), for prevalent CVD OR 2.22 (95% CI 1.87–2.62), cancer OR 2.05 (95% CI 1.73–2.42) and diabetes OR 3.41 (95% CI 2.74–4.24). The risk of multimorbidity in participants with CVD at baseline is equivalent to the risk in those without CVD 10 years older. Similarly, in participants with baseline diabetes and baseline cancer, the risk equivalent to those without disease is 17 and 11 years older respectively.

Table 3 | Multivariable logistic regression of risk factors for Charlson 5-year and 10-year hospital admissions with multimorbidity in 25014 men and women

	Charlson 5-year multimorbidity †, 1999–2004 OR (95% CI)	p value	Charlson 10-year multimorbidity †, 1999–2009 OR (95% CI)	p value
**Model 1**				
Male sex	1.49 (1.34–1.67)	< 0.001	1.56 (1.44–1.69)	< 0.001
Age per 10 years	2.27 (2.13–2.44)	< 0.001	2.34 (2.23–2.46)	< 0.001
Manual social class	1.20 (1.07–1.35)	0.002	1.22 (1.12–1.33)	< 0.001
Lower education level	1.15 (1.02–1.30)	0.023	1.19 (1.09–1.30)	< 0.001
**Model 2**				
Male sex	1.39 (1.24–1.56)	< 0.001	1.47 (1.35–1.60)	< 0.001
Age per 10 years	2.11 (1.97–2.26)	< 0.001	2.21 (2.10–2.32)	< 0.001
Manual social class	1.22 (1.08–1.37)	0.001	1.23 (1.13–1.34)	< 0.001
Lower education level	1.13 (1.00–1.28)	0.053	1.17 (1.07–1.28)	< 0.001
Prevalent CVD	2.23 (1.85–2.68)	< 0.001	2.25 (1.93–2.60)	< 0.001
Prevalent cancer	2.11 (1.75–2.54)	< 0.001	1.92 (1.65–2.22)	< 0.001
Prevalent diabetes	4.41 (3.55–5.45)	< 0.001	4.32 (3.57–5.21)	< 0.001
**Model 3**				
Male sex	1.24 (1.07–1.42)	0.003	1.33 (1.20–1.47)	< 0.001
Age per 10 years	2.16 (1.99–2.34)	< 0.001	2.29 (2.16–2.43)	< 0.001
Manual social class	1.09 (0.95–1.25)	0.214	1.17 (1.06–1.29)	0.002
Lower education level	1.06 (0.92–1.21)	0.447	1.08 (0.98–1.20)	0.112
Current smoker	1.71 (1.42–2.05)	< 0.001	1.73 (1.51–1.98)	< 0.001
BMI>30 kg/m²	1.32 (1.12–1.56)	< 0.001	1.45 (1.28–1.63)	< 0.001
Alcohol intake, units per week	1.00 (0.99–1.01)	0.872	1.00 (1.00–1.01)	0.666
Physically inactive	1.26 (1.10–1.44)	< 0.001	1.15 (1.04–1.26)	0.006
Plasma vitamin C per SD	0.81 (0.75–0.86)	< 0.001	0.84 (0.80–0.88)	< 0.001
Prevalent CVD	2.02 (1.63–2.49)	< 0.001	2.17 (1.84–2.57)	< 0.001
Prevalent cancer	2.22 (1.79–2.72)	< 0.001	2.06 (1.74–2.43)	< 0.001
Prevalent diabetes	3.53 (2.73–4.52)	< 0.001	3.54 (2.85–4.39)	< 0.001
**Model 4**				

For peer review only - http://bmjopen.bmj.com/site/about/guidelines.xhtml

	Charlson 5-year multimorbidity †, 1999–2004 OR (95% CI)	p value	Charlson 10-year multimorbidity †, 1999–2009 OR (95% CI)	p value
Male sex	1.23 (1.07–1.43)	0.005	1.32 (1.19–1.47)	< 0.001
Age per 10 years	2.08 (1.91–2.27)	< 0.001	2.19 (2.06–2.33)	< 0.001
Manual social class	1.09 (0.95–1.25)	0.235	1.16 (1.05–1.28)	0.004
Lower education level	1.06 (0.92–1.22)	0.420	1.09 (0.99–1.21)	0.091
Current smoker	1.72 (1.43–2.07)	< 0.001	1.74 (1.52–2.00)	< 0.001
BMI>30 kg/m²	1.31 (1.11–1.54)	0.001	1.40 (1.24–1.58)	< 0.001
Alcohol intake, units per week	1.00 (0.99–1.01)	0.878	1.00 (1.00–1.01)	0.800
Physically inactive	1.25 (1.09–1.43)	0.001	1.14 (1.04–1.26)	0.008
Plasma vitamin C per SD	0.81 (0.76–0.87)	< 0.001	0.85 (0.81–0.89)	< 0.001
Systolic blood pressure per SD	1.10 (1.03–1.17)	0.005	1.12 (1.07–1.18)	< 0.001
Total cholesterol per SD	0.99 (0.92–1.05)	0.690	0.99 (0.94–1.04)	0.614
Prevalent CVD	2.06 (1.66–2.54)	< 0.001	2.22 (1.87–2.62)	< 0.001
Prevalent cancer	2.23 (1.80–2.75)	< 0.001	2.05 (1.73–2.42)	< 0.001
Prevalent diabetes	3.42 (2.64–4.39)	< 0.001	3.41 (2.74–4.24)	< 0.001

† Charlson Comorbidity Index ≥3 vs Charlson ≤2 or no hospital admission.

The models in table 4 are similar to those used in table 3 but rather than adjusting for prevalent disease, participants who reported heart attack, stroke, cancer or diabetes at baseline were excluded. In this subgroup of participants without known common major diseases, in addition to age and sex, current cigarette smoking OR 1.74 (95% CI 1.52–2.00), BMI >30 kg/m^2 OR 1.40 (95% CI 1.24–1.58) and physical inactivity OR 1.14 (95% CI 1.04–1.26) were positively associated and plasma vitamin C OR 0.85 (95% CI 0.81–0.89) inversely associated with incident 10-year hospital admissions with multimorbidity after multivariable adjustment for age, sex, social class, education, alcohol consumption, systolic blood pressure and cholesterol (model 3). Manual social class and educational attainment were associated with incident multimorbidity in model 1 but were attenuated in model 2 and 3. An inverse association was observed for total cholesterol while systolic blood pressure appeared to be associated but the direction of association was not consistent with the repeated analyses from TP2. There was no association for alcohol in these models. The risk of multimorbidity in current cigarette smokers is equivalent to the risk in non-smokers 7 years older, while each 20 μmol/L rise in plasma vitamin C (approximately two servings of fruit and vegetables per day [38]) corresponds to a reduction in risk equivalent risk of those 3 years younger.

Table 4 | Multivariable logistic regression of risk factors excluding participants with prevalent CVD, cancer or diabetes for Charlson 5-year and 10-year hospital admissions with multimorbidity in 22278 men and women

	Charlson 5-year multimorbidity †, 1999–2004 OR (95% CI)	p value	Charlson 10-year multimorbidity †, 1999–2009 OR (95% CI)	p value
**Model 1**				
Male sex	1.47 (1.29–1.68)	< 0.001	1.52 (1.38–1.67)	< 0.001
Age per 10 years	2.19 (2.02–2.37)	< 0.001	2.31 (2.19–2.45)	< 0.001
Manual social class	1.23 (1.07–1.42)	0.003	1.22 (1.11–1.34)	< 0.001
Lower education level	1.20 (1.04–1.39)	0.011	1.16 (1.05–1.28)	0.003
**Model 2**				
Male sex	1.32 (1.13–1.55)	< 0.001	1.39 (1.24–1.55)	< 0.001
Age per 10 years	2.24 (2.05–2.46)	< 0.001	2.40 (2.25–2.56)	< 0.001
Manual social class	1.13 (0.96–1.32)	0.131	1.17 (1.05–1.30)	0.006

For peer review only - http://bmjopen.bmj.com/site/about/guidelines.xhtml
For peer review only

	Charlson 5-year multimorbidity †, 1999–2004 OR (95% CI)	p value	Charlson 10-year multimorbidity †, 1999–2009 OR (95% CI)	p value
Lower education level	1.07 (0.91–1.25)	0.416	1.05 (0.93–1.17)	0.428
Current smoker	1.85 (1.50–2.26)	< 0.001	1.84 (1.58–2.13)	< 0.001
BMI>30 kg/m²	1.31 (1.07–1.58)	0.006	1.53 (1.34–1.75)	< 0.001
Alcohol intake, units per week	1.00 (0.99–1.01)	0.789	1.00 (1.00–1.01)	0.805
Physically inactive	1.25 (1.07–1.46)	0.004	1.17 (1.05–1.31)	0.005
Plasma vitamin C per SD	0.82 (0.76–0.89)	< 0.001	0.85 (0.80–0.90)	< 0.001
**Model 3**				
Male sex	1.32 (1.12–1.56)	0.001	1.37 (1.22–1.54)	< 0.001
Age per 10 years	2.15 (1.95–2.37)	< 0.001	2.30 (2.15–2.46)	< 0.001
Manual social class	1.11 (0.95–1.31)	0.178	1.15 (1.03–1.29)	0.012
Lower education level	1.07 (0.91–1.26)	0.383	1.05 (0.94–1.18)	0.393
Current smoker	1.88 (1.52–2.30)	< 0.001	1.86 (1.60–2.15)	< 0.001
BMI>30 kg/m²	1.30 (1.07–1.58)	0.007	1.48 (1.30–1.70)	< 0.001
Alcohol intake, units per week	1.00 (0.99–1.01)	0.828	1.00 (0.99–1.01)	0.941
Physically inactive	1.24 (1.06–1.45)	0.007	1.16 (1.04–1.29)	0.009
Plasma vitamin C per SD	0.83 (0.77–0.90)	< 0.001	0.86 (0.81–0.91)	< 0.001
Systolic blood pressure per SD	1.12 (1.03–1.21)	0.005	1.13 (1.07–1.19)	< 0.001
Total cholesterol per SD	0.98 (0.91–1.06)	0.607	0.97 (0.92–1.03)	0.328

† Charlson Comorbidity Index ≥3 vs Charlson ≤2 or no hospital admission.

For peer review only

Supplementary table S1 shows the descriptive characteristics of participants at TP2 for 10-year CCI. Mean age in this subset, measured approximately 12 years after baseline, was 69.4. Hospital admissions and length of stay was similar to the baseline with multimorbid participants (CCI ≥3) having much longer duration than those with non-multimorbid participants or those who had no hospital admissions. Multimorbid participants were inactive, had lower plasma vitamin C (reflecting a lower intake of fruit and vegetables), were current or former smokers and had prevalent disease. In supplementary table S2, multivariable models of 10-year incident multimorbidity show that prevalent diabetes, CVD and cancer are all strongly associated. After excluding prevalent disease, supplementary table S3 shows multivariate associations in a group free from the most serious diseases at TP2. Both age and male sex are associated with subsequent multimorbidity, with educational attainment, current cigarette smoking, plasma vitamin C, BMI>30 kg/m^2 and physical inactivity all predicting future multimorbidity. Systolic blood pressure was attenuated while other factors including cholesterol were more strong associated than at baseline. Supplementary table S4 shows the ICD-10 codes corresponding to Charlson Comorbidity Index disease groups.

Discussion

In this community-based population followed prospectively, we observed incident hospital multimorbidity admissions rates over 5-year and 10-years periods which, as expected were strongly related to increasing age. We also observed that those with multimorbid hospital admissions had substantially more days in hospital over the outcome periods. In multivariable analyses, risk of such admissions is predicted by age, male sex and several potentially modifiable factors. Participants at baseline who smoked cigarettes, had BMI >30, were physically inactive or had a diet low in fruit and

vegetables all had higher likelihood of having subsequent hospital admissions with multimorbidity. Measurements made on a subset of the cohort 12 years after baseline who were followed up subsequently confirmed the baseline findings while also demonstrating an association for low education level in an older cohort with incident multimorbidity.

Strengths and limitations of study

Most studies of multimorbidity focus on its consequences and those examining risk factors for multimorbidity are largely cross-sectional. While many prospective studies have examined the relationship between baseline characteristics and specific incident diseases or mortality, establishing multimorbidity as an endpoint is more challenging. By using the Charlson Comorbidity Index to define multimorbidity, we were able to show that the chronic diseases defined by the index had considerably higher average length of stay than other conditions requiring hospitalisation and that length of stay increased with higher CCI score. The current population-based study in a defined community was able to assess incident hospital admissions with multimorbidity to enable estimates of 5-year and 10-year rates by age and sex. We were also able to document the relationship between demographic, lifestyle and physiological factors and subsequent hospitalisations for multimorbidity. The EPIC-Norfolk cohort has been followed for 20 years enabling us to examine the determinants of multimorbidity at two time-points: in mainly middle-aged participants of 40–79 years and mainly old-aged participants of 48–92 years in a sub-cohort 12 years later after major organisational changes had been made to the National Health Service (NHS). We were also able to examine associations with and without excluding participants with known prevalent conditions at baseline.

While not attempting to examine clusters or pathways of chronic disease, we have identified risk factors that predict any hospital admissions with multimorbidity. It is possible that some factors we observed will be more strongly associated with certain combinations of diseases and others less so. However, the burden of resources experienced by hospitals can best be mitigated by early public health advice, prior to the onset of disease if possible, which can only be general in nature. Our findings are in line with current public health advice such as smoking cessation, a diet containing fruit and vegetables and regular exercise and, given the huge additional burden placed on the NHS by multimorbidity, should further emphasise the need for public health advice and intervention.

Multimorbidity can be defined in a number of ways such as disease counts or using various indexes. [39] By restricting the definition to a relatively small subset of chronic conditions such as in the CCI, inevitably some conditions will not be counted. It is notable that the CCI does not include depression or mental health, asthma or respiratory diseases, epilepsy, hypothyroidism, musculoskeletal problems or atrial fibrillation, all common in a primary care setting. [40] In addition to the CCI and other commonly used systems [41], authors have used many other definitions with variable numbers of

For peer review only - http://bmjopen.bmj.com/site/about/guidelines.xhtml

underlying conditions and hence the prevalence of multimorbidity varies widely. However, CCI is a widely used measure of multimorbidity.

Since the CCI is weighted to predict mortality, it may be better able to assess health service burden than a simple disease count, since procedures required for higher weighted conditions will generally be more costly. However, it may be less effective as an indicator of multiple long-term conditions. Some chronic conditions such as musculoskeletal and mental health diseases not included in the CCI are nevertheless likely to require long stay inpatient care. However, increasing CCI had longer hospital length of stay in the present study and this has also been reported in several other studies. [42,43] Medical conditions such as obesity have well established links to many diseases but, as non-diseases, are not included in the CCI. The use of CCI ≥3 to define multimorbidity classifies a small number of participants with one serious disease with a high CCI weight as multimorbid. However, a sensitivity excluding these people gave virtually identical results. Studies examining the longitudinal predictors of future multimorbidity generally rely on self-reported disease but our study used the CCI from linked hospital medical coding.

When examining the relationship between lifestyle factors and health outcomes, confounding will always be a limitation. Individuals who smoke, are less physically active and eat a poor diet for example, are likely to differ from those with a contrasting lifestyle with respect to other factors relating to the likelihood of future multimorbidity including their age, sex, lifestyle factors examined in this study and others unknown. However, the associations we report were consistent after multivariable adjustment for other factors. Differential mortality is another possible limitation and would occur for any of the factors examined if participants with an apparently unhealthy characteristic were more likely to have died earlier than those with the contrary healthy characteristic and hence were less likely to use hospital services for the full follow-up period. However, the results for the 5-year follow-up period where very few deaths occurred were consistent with the longer 10-year follow-up period. While it is possible that some participants were multimorbid at baseline, we examined those with and without baseline self-reported major chronic disease.

Comparison with other studies

Estimates of the prevalence of multimorbidity vary widely, partly due to the variety of definitions, number of diseases, weighting etc. used in studies but range from 55 to 98% in the elderly .[6] Most studies report multimorbidity associated with age and present in more than half of those aged 65 and older. [3,44] Age was strongly associated with future hospitalisation and incident multimorbidity in our study and has been reported to increase hospitalised multimorbidity in elderly patients. [45] Many studies have found that women have a higher rate of multimorbidity than men [6,44,46–48], but we observed the converse with male sex strongly predicting future multimorbidity. The use of CCI in the

For peer review only - http://bmjopen.bmj.com/site/about/guidelines.xhtml

context of prospective hospital admissions rather than cross-sectional multimorbidity in a primary care setting may explain the higher proportion of multimorbid men. Physical-mental comorbidity is reported higher among women in primary care [49] and mental health, which is not included in the CCI, may be more likely to be treated in a primary care than in an acute hospital setting.

Despite the considerable literature relating to multimorbidity, very few studies have examined the modifiable determinants of incident multimorbidity. Incident Cancer and cardiometabolic multimorbidity was examined in a recent multi-centre study which included data from the present study [21]; pre-diagnostic healthy lifestyle behaviours were reported to be inversely associated with the risk. BMI was also reported to be associated with incident cardiometabolic multimorbidity in a pooled analysis of 16 cohort studies. [22] A Finnish study examined incident multimorbidity in both disease-free and those with baseline diabetes and CVD. [24] They reported some similar findings to the present study such as associations with cigarette smoking, physical inactivity and BMI but associations for low education level and systolic blood pressure were only found in men. Multimorbidity was defined as the five common diseases and time to event 10-year follow-up was used rather than a follow-up period approach in this study. Participants in the Finnish cohort were younger than those in EPIC-Norfolk with the oldest participant 74 years at the end of follow-up against 90 years in EPIC-Norfolk baseline and 100 years at TP2. Studies using data from an English longitudinal cohort and using self-reported disease counts to define multimorbidity reported associations in physical activity, obesity and low level of wealth and an increased risk of multimorbidity when combined with other lifestyle factors such smoking, obesity and inadequate fruit and vegetable consumption. [25,26] However, they found no association with educational attainment or excess alcohol consumption. Education, which was associated in older participants at TP2 in our study, has been linked to multimorbidity in cross-sectional studies [50] and prospectively. [24] Socioeconomic status was reported to predict the development of multimorbidity throughout the life course in a Scottish longitudinal study. [51] Both educational attainment and occupational social class were attenuated in our study possibly due to the models including plasma vitamin C, also a marker of socioeconomic status. While smoking was a strong predictor, we did not find an association with alcohol drinking. However, other studies in the literature are inconsistent, with some finding no association with cigarette smoking and alcohol consumption in cross-sectional analyses [1]

Generalisability

While hospital admissions with multimorbidity provide an objective indicator of both Health Service and individual burden of the condition, studies of hospital admissions in many countries are limited by factors relating to differential accessibility to health care such as health insurance, income and health care policy. Though not entirely free of differential accessibility, the NHS in the UK, with service free at the point of delivery for all residents, provides an opportunity to examine hospitalised multimorbidity

For peer review only - http://bmjopen.bmj.com/site/about/guidelines.xhtml

with fewer of these constraints. Health care policy and criteria for admission change over time, not least in the UK over the 20-year period of this study, so we examined admissions and risk factors for multimorbidity over two independent time periods using new repeated measures and found consistent results.

Conclusions and policy implications

We observed in a long-term population-based study that age, male sex and potentially modifiable factors including smoking, body mass index, physical inactivity and a diet low in fruit and vegetables predict future incident hospitalised multimorbidity. Multimorbidity is increasingly common among elderly hospital inpatients due in part to improved efficacy of treatments and drugs. While considerable effort is being focused on the progression, disease clustering and treatment of patients with multimorbidity, there has been less attention on the long-term predictors of future incident multimorbidity. This study suggests that modest difference in lifestyles may have the potential to mitigate the future burden of multimorbidity in the population.

For peer review only

For peer review only - http://bmjopen.bmj.com/site/about/guidelines.xhtml

References

1 The Academy of Medical Sciences. Multimorbidity: a priority for global health research | The Academy of Medical Sciences. 2018. https://acmedsci.ac.uk/policy/policy-projects/multimorbidity

2 King DE, Xiang J, Pilkerton CS. Multi-morbidity Trends in United States adults, 1988–2014. *J Am Board Fam Med* 2018;**31**:503–13. doi:10.3122/jabfm.2018.04.180008

3 Barnett K, Mercer SW, Norbury M, *et al.* Epidemiology of multimorbidity and implications for health care, research, and medical education: a cross-sectional study. *The Lancet* 2012;**380**:37–43. doi:10.1016/S0140-6736(12)60240-2

4 Johnston MC, Crilly M, Black C, *et al.* Defining and measuring multimorbidity: a systematic review of systematic reviews. *Eur J Public Health* 2019;**29**:182–9. doi:10.1093/eurpub/cky098

5 van den Akker M, Buntinx F, Knottnerus JA. Comorbidity or multimorbidity. *European Journal of General Practice* 1996;**2**:65–70. doi:10.3109/13814789609162146

6 Marengoni A, Angleman S, Melis R, *et al.* Aging with multimorbidity: A systematic review of the literature. *Ageing Research Reviews* 2011;**10**:430–9. doi:10.1016/j.arr.2011.03.003

7 Feinstein AR. THE PRE-THERAPEUTIC CLASSIFICATION OF CO-MORBIDITY IN CHRONIC DISEASE. *J Chronic Dis* 1970;**23**:455–68. doi:10.1016/0021-9681(70)90054-8

8 Charlson ME, Pompei P, Ales KL, *et al.* A new method of classifying prognostic comorbidity in longitudinal studies: Development and validation. *Journal of Chronic Diseases* 1987;**40**:373–83. doi:10.1016/0021-9681(87)90171-8

9 Romano PS, Roos LL, Jollis JG. Presentation adapting a clinical comorbidity index for use with ICD-9-CM administrative data: Differing perspectives. *Journal of Clinical Epidemiology* 1993;**46**:1075–9. doi:10.1016/0895-4356(93)90103-8

10 Groot V de, Beckerman H, Lankhorst GJ, *et al.* How to measure comorbidity: a critical review of available methods. *Journal of Clinical Epidemiology* 2003;**56**:221–9. doi:10.1016/S0895-4356(02)00585-1

11 Extermann M. Measurement and impact of comorbidity in older cancer patients. *Critical Reviews in Oncology/Hematology* 2000;**35**:181–200. doi:10.1016/S1040-8428(00)00090-1

12 Quan H, Sundararajan V, Halfon P, *et al.* Coding Algorithms for Defining Comorbidities in ICD-9-CM and ICD-10 Administrative Data: *Medical Care* 2005;**43**:1130–9. doi:10.1097/01.mlr.0000182534.19832.83

13 Menendez ME, Neuhaus V, van Dijk CN, *et al.* The Elixhauser Comorbidity Method Outperforms the Charlson Index in Predicting Inpatient Death After Orthopaedic Surgery. *Clin Orthop Relat Res* 2014;**472**:2878–86. doi:10.1007/s11999-014-3686-7

14 Quan H, Li B, Couris CM, *et al.* Updating and Validating the Charlson Comorbidity Index and Score for Risk Adjustment in Hospital Discharge Abstracts Using Data From 6 Countries. *Am J Epidemiol* 2011;**173**:676–82. doi:10.1093/aje/kwq433

15 Sharabiani MTA, Aylin P, Bottle A. Systematic Review of Comorbidity Indices for Administrative Data: *Medical Care* 2012;**50**:1109–18. doi:10.1097/MLR.0b013e31825f64d0

For peer review only - http://bmjopen.bmj.com/site/about/guidelines.xhtml

16 Caughey GE, Ramsay EN, Vitry AI, *et al.* Comorbid chronic diseases, discordant impact on mortality in older people: a 14-year longitudinal population study. *Journal of Epidemiology & Community Health* 2010;**64**:1036–42. doi:10.1136/jech.2009.088260

17 Lund Jensen N, Pedersen HS, Vestergaard M, *et al.* The impact of socioeconomic status and multimorbidity on mortality: a population-based cohort study. *Clin Epidemiol* 2017;**9**:279–89. doi:10.2147/CLEP.S129415

18 Stanley J, Sarfati D. The new measuring multimorbidity index predicted mortality better than Charlson and Elixhauser indices among the general population. *Journal of Clinical Epidemiology* 2017;**92**:99–110. doi:10.1016/j.jclinepi.2017.08.005

19 Teh RO, Menzies OH, Connolly MJ, *et al.* Patterns of multi-morbidity and prediction of hospitalisation and all-cause mortality in advanced age. *Age Ageing* 2018;**47**:261–8. doi:10.1093/ageing/afx184

20 Nunes BP, Flores TR, Mielke GI, *et al.* Multimorbidity and mortality in older adults: A systematic review and meta-analysis. *Archives of Gerontology and Geriatrics* 2016;**67**:130–8. doi:10.1016/j.archger.2016.07.008

21 Freisling H, Viallon V, Lennon H, *et al.* Lifestyle factors and risk of multimorbidity of cancer and cardiometabolic diseases: a multinational cohort study. *BMC Medicine* 2020;**18**:5. doi:10.1186/s12916-019-1474-7

22 Kivimäki M, Kuosma E, Ferrie JE, *et al.* Overweight, obesity, and risk of cardiometabolic multimorbidity: pooled analysis of individual-level data for 120 813 adults from 16 cohort studies from the USA and Europe. *The Lancet Public Health* 2017;**2**:e277–85. doi:10.1016/S2468-2667(17)30074-9

23 Gijsen R, Hoeymans N, Schellevis FG, *et al.* Causes and consequences of comorbidity: A review. *Journal of Clinical Epidemiology* 2001;**54**:661–74. doi:10.1016/S0895-4356(00)00363-2

24 Wikström K, Lindström J, Harald K, *et al.* Clinical and lifestyle-related risk factors for incident multimorbidity: 10-year follow-up of Finnish population-based cohorts 1982–2012. *European Journal of Internal Medicine* 2015;**26**:211–6. doi:10.1016/j.ejim.2015.02.012

25 Dhalwani NN, Zaccardi F, O'Donovan G, *et al.* Association Between Lifestyle Factors and the Incidence of Multimorbidity in an Older English Population. *J Gerontol A Biol Sci Med Sci* 2017;**72**:528–34. doi:10.1093/gerona/glw146

26 Mounce LTA, Campbell JL, Henley WE, *et al.* Predicting Incident Multimorbidity. *Ann Fam Med* 2018;**16**:322–9. doi:10.1370/afm.2271

27 Luben R, Hayat S, Wareham N, *et al.* Usual physical activity and subsequent hospital usage over 20 years in a general population: the EPIC-Norfolk cohort. *BMC Geriatrics* 2020;**20**:165. doi:10.1186/s12877-020-01573-0

28 Luben R, Hayat S, Khawaja A, *et al.* Residential area deprivation and risk of subsequent hospital admission in a British population: the EPIC-Norfolk cohort. *BMJ Open* 2019;**9**. doi:10.1136/bmjopen-2019-031251

29 Luben R, Hayat S, Wareham N, *et al.* Predicting admissions and time spent in hospital over a decade in a population-based record linkage study: the EPIC-Norfolk cohort. *BMJ Open* 2016;**6**:e009461. doi:10.1136/bmjopen-2015-009461

For peer review only - http://bmjopen.bmj.com/site/about/guidelines.xhtml

30 Day N, Oakes S, Luben R, *et al.* EPIC-Norfolk: study design and characteristics of the cohort. European Prospective Investigation of Cancer. *Br J Cancer* 1999;**80 Suppl 1**:95–103.

31 Hayat SA, Luben R, Keevil VL, *et al.* Cohort Profile: A prospective cohort study of objective physical and cognitive capability and visual health in an ageing population of men and women in Norfolk (EPIC-Norfolk 3). *Int J Epidemiol* 2014;**43**:1063–72. doi:10.1093/ije/dyt086

32 Vuilleumier JP, Keck E. Fluorometric assay of vitamin C in biological materials using a centrifugal analyser with fluorescence attachment. *Journal of micronutrient analysis* Published Online First: 1989.http://agris.fao.org/agris-search/search.do?recordID=US201301738860

33 Bland R. Measuring "Social Class": A Discussion of the Registrar-General's Classification. *Sociology* 1979;**13**:283–91. doi:10.1177/003803857901300209

34 McFadden E, Luben R, Khaw K-T. Different measures of social class in women and mortality. *Eur J Epidemiol* 2009;**24**:231–6. doi:10.1007/s10654-009-9332-0

35 Wareham NJ, Jakes RW, Rennie KL, *et al.* Validity and repeatability of a simple index derived from the short physical activity questionnaire used in the European Prospective Investigation into Cancer and Nutrition (EPIC) study. *Public Health Nutrition* 2003;**6**:407–13. doi:10.1079/PHN2002439

36 Khaw K-T, Jakes R, Bingham S, *et al.* Work and leisure time physical activity assessed using a simple, pragmatic, validated questionnaire and incident cardiovascular disease and all-cause mortality in men and women: The European Prospective Investigation into Cancer in Norfolk prospective population study. *Int J Epidemiol* 2006;**35**:1034–43. doi:10.1093/ije/dyl079

37 Gasparini A. comorbidity: An R package for computing comorbidity scores. *JOSS* 2018;**3**:648. doi:10.21105/joss.00648

38 Khaw K-T, Wareham N, Bingham S, *et al.* Combined Impact of Health Behaviours and Mortality in Men and Women: The EPIC-Norfolk Prospective Population Study. *PLOS Medicine* 2008;**5**:e12. doi:10.1371/journal.pmed.0050012

39 Wallace E, McDowell R, Bennett K, *et al.* Comparison of count-based multimorbidity measures in predicting emergency admission and functional decline in older community-dwelling adults: a prospective cohort study. *BMJ Open* 2016;**6**. doi:10.1136/bmjopen-2016-013089

40 Department of Health UK. Long Term Conditions Compendium of Information: Third Edition. 2012. https://www.gov.uk/government/publications/long-term-conditions-compendium-of-information-third-edition

41 Elixhauser A, Steiner C, Harris DR, *et al.* Comorbidity Measures for Use with Administrative Data: *Medical Care* 1998;**36**:8–27. doi:10.1097/00005650-199801000-00004

42 Librero J, Peiró S, Ordiñana R. Chronic Comorbidity and Outcomes of Hospital Care: Length of Stay, Mortality, and Readmission at 30 and 365 Days. *Journal of Clinical Epidemiology* 1999;**52**:171–9. doi:10.1016/S0895-4356(98)00160-7

43 Palladino R, Tayu Lee J, Ashworth M, *et al.* Associations between multimorbidity, healthcare utilisation and health status: evidence from 16 European countries. *Age Ageing* 2016;**45**:431–5. doi:10.1093/ageing/afw044

44 Nguyen H, Manolova G, Daskalopoulou C, *et al.* Prevalence of multimorbidity in community settings: A

For peer review only

systematic review and meta-analysis of observational studies. *J Comorb* 2019;**9**:2235042X1987093. doi:10.1177/2235042X19870934

45 Lai FTT, Wong SYS, Yip BHK, *et al.* Multimorbidity in middle age predicts more subsequent hospital admissions than in older age: A nine-year retrospective cohort study of 121,188 discharged in-patients. *European Journal of Internal Medicine* 2019;**61**:103–11. doi:10.1016/j.ejim.2018.12.001

46 Violán C, Foguet-Boreu Q, Roso-Llorach A, *et al.* Burden of multimorbidity, socioeconomic status and use of health services across stages of life in urban areas: a cross-sectional study. *BMC Public Health* 2014;**14**:530. doi:10.1186/1471-2458-14-530

47 Chung RY, Mercer S, Lai FTT, *et al.* Socioeconomic Determinants of Multimorbidity: A Population-Based Household Survey of Hong Kong Chinese. *PLOS ONE* 2015;**10**:e0140040. doi:10.1371/journal.pone.0140040

48 Alimohammadian M, Majidi A, Yaseri M, *et al.* Multimorbidity as an important issue among women: results of a gender difference investigation in a large population-based cross-sectional study in West Asia. *BMJ Open* 2017;**7**. doi:10.1136/bmjopen-2016-013548

49 Cassell A, Edwards D, Harshfield A, *et al.* The epidemiology of multimorbidity in primary care: a retrospective cohort study. *Br J Gen Pract* 2018;**68**:e245–51. doi:10.3399/bjgp18X695465

50 Nagel G, Peter R, Braig S, *et al.* The impact of education on risk factors and the occurrence of multimorbidity in the EPIC-Heidelberg cohort. *BMC Public Health* 2008;**8**:384. doi:10.1186/1471-2458-8-384

51 Katikireddi SV, Skivington K, Leyland AH, *et al.* The contribution of risk factors to socioeconomic inequalities in multimorbidity across the lifecourse: a longitudinal analysis of the Twenty-07 cohort. *BMC Med* 2017;**15**. doi:10.1186/s12916-017-0913-6

For peer review only

For peer review only - http://bmjopen.bmj.com/site/about/guidelines.xhtml

Supplementary material

Supplementary Table S1 | Descriptive characteristics at TP2 in 9814 men and women aged 48–92 by 10-year Charlson Comorbidity Index, 2009–2019

Supplementary Table S2 | Multivariable logistic regression of risk factors for Charlson 5-year and 10-year hospital admissions with multimorbidity at TP2 in 9814 men and women

Supplementary Table S3 | Multivariable logistic regression of risk factors excluding participants with prevalent CVD, cancer or diabetes at TP2 for Charlson 5-year and 10-year hospital admissions with multimorbidity at TP2 in 8185 men and women

Supplementary Table S4 | Charlson Comorbidity Index, ICD-10 codes and weighting

For peer review only

For peer review only - http://bmjopen.bmj.com/site/about/guidelines.xhtml

For peer review only

For peer review only - http://bmjopen.bmj.com/site/about/guidelines.xhtml

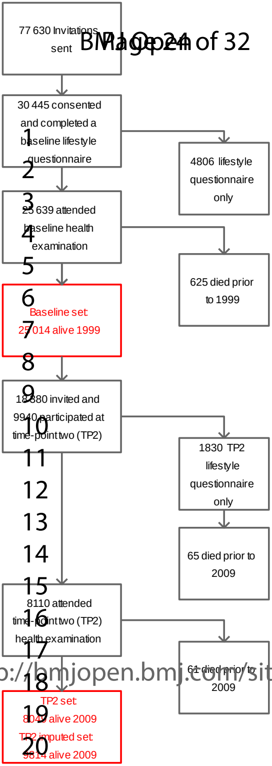

http://bmjopen.bmj.com/sit

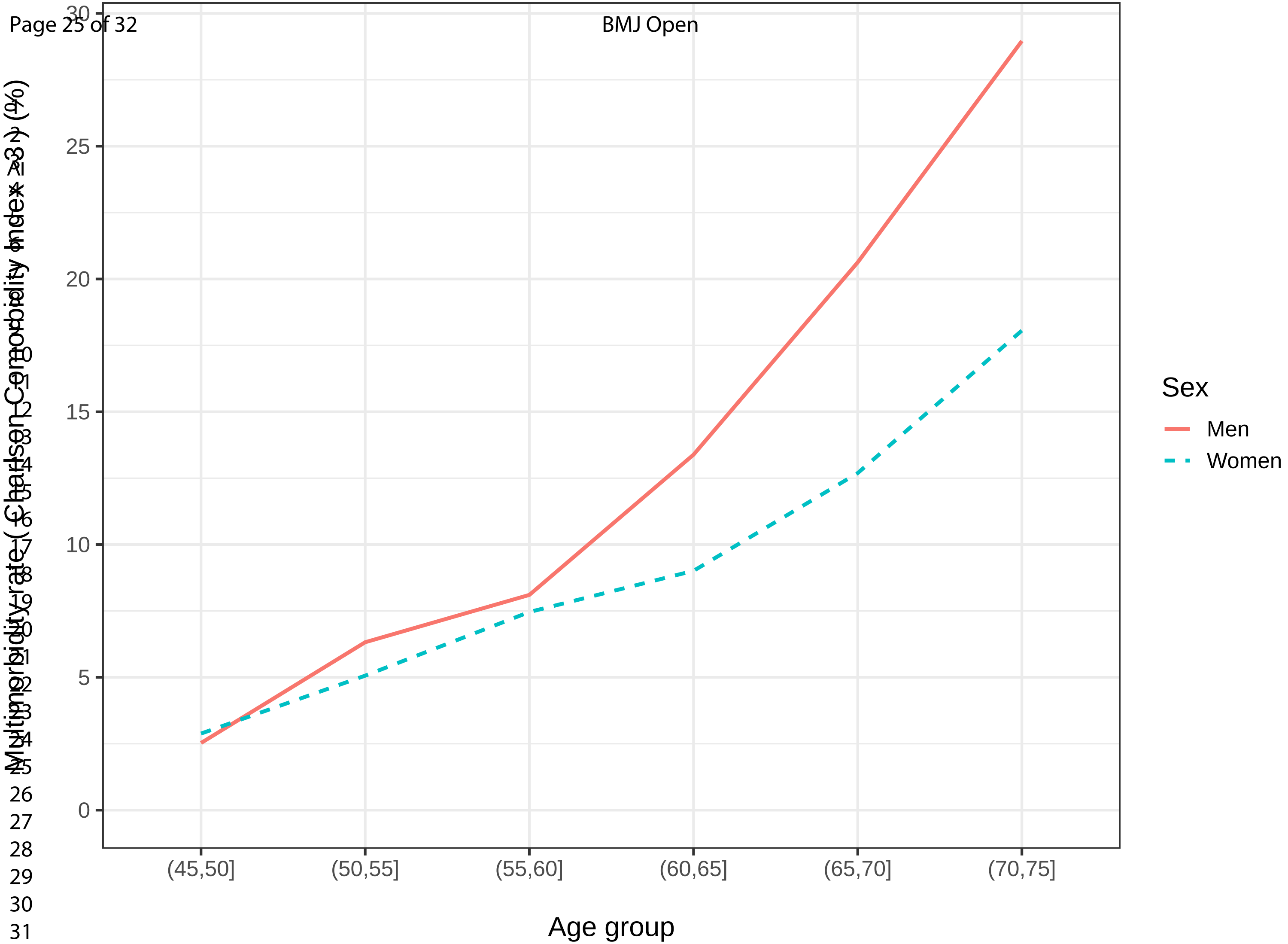
Multimorbidity rate (Charlson Comorbidity Index ≥ 3) (%)
30
25
20
15
10
5
0
(45,50]
(50,55]
(55,60]
(60,65]
(65,70]
(70,75]
Age group
Sex
Men
Women

Sociodemographic and lifestyle predictors of incident hospital admissions with multimorbidity in a general population 1999–2019: the EPIC-Norfolk cohort

Supplementary material

Supplementary Table S1 | Descriptive characteristics at TP2 in 9814 men and women aged 48–92 by 10-year Charlson Comorbidity Index, 2009–2019

Supplementary Table S2 | Multivariable logistic regression of risk factors for Charlson 5-year and 10-year hospital admissions with multimorbidity at TP2 in 9814 men and women

Supplementary Table S3 | Multivariable logistic regression of risk factors excluding participants with prevalent CVD, cancer or diabetes at TP2 for Charlson 5-year and 10-year hospital admissions with multimorbidity at TP2 in 8185 men and women

Supplementary Table S4 | Charlson Comorbidity Index, ICD-10 codes and weighting

For peer review only

Supplementary Table S1 | Descriptive characteristics at TP2 in 9814 men and women aged 48–92 by 10-year Charlson Comorbidity Index, 2009–2019

	Total	No admissions	Charlson 0	Charlson 1	Charlson 2	Charlson ≥3
**Hospital duration 2009–2019, days**						
Mean ±SD	17.7 ±36.2	0.0 ±0.0	6.5 ±14.0	19.3 ±42.8	26.1 ±42.2	46.3 ±48.1
**Total hospital admissions 2009–2019**						
Mean ±SD	4.4 ±7.9	0.0 ±0.0	2.8 ±3.0	4.5 ±6.0	6.0 ±6.7	10.1 ±13.5
**Age at TP2, years**						
Mean ±SD	69.4 ±8.4	65.1 ±7.7	67.6 ±7.7	70.5 ±8.1	71.7 ±8.1	74.1 ±7.8
**Sex (n (%))**						
Men	4252	695 (16.3)	1294 (30.4)	631 (14.8)	558 (13.1)	1074 (25.3)
Women	5562	1166 (21.0)	1956 (35.2)	914 (16.4)	618 (11.1)	908 (16.3)
**Body mass index at TP2, kg/m²**						
Mean ±SD	26.9 ±4.3	26.2 ±4.3	26.5 ±4.0	27.3 ±4.4	27.3 ±4.5	27.7 ±4.5
**Cigarette smoking at TP2 (n (%))**						
Current	442	88 (19.9)	116 (26.2)	65 (14.7)	79 (17.9)	94 (21.3)
Former	4508	755 (16.7)	1375 (30.5)	741 (16.4)	565 (12.5)	1072 (23.8)
Never	4864	1018 (20.9)	1759 (36.2)	739 (15.2)	532 (10.9)	816 (16.8)
**Social class dichotomised (n (%))**						
Non-manual	6294	1205 (19.1)	2127 (33.8)	993 (15.8)	749 (11.9)	1220 (19.4)
Manual	3411	636 (18.6)	1087 (31.9)	528 (15.5)	424 (12.4)	736 (21.6)
**Level of education (n (%))**						
Higher level	7025	1460 (20.8)	2419 (34.4)	1074 (15.3)	791 (11.3)	1281 (18.2)
Lower level	2787	401 (14.4)	830 (29.8)	471 (16.9)	385 (13.8)	700 (25.1)
**Simple physical activity index at TP2 (n (%))**						
Inactive	3924	592 (15.1)	1072 (27.3)	687 (17.5)	545 (13.9)	1028 (26.2)
Moderately inactive	2682	555 (20.7)	940 (35.0)	404 (15.1)	311 (11.6)	472 (17.6)
Moderately active	1654	387 (23.4)	604 (36.5)	231 (14.0)	167 (10.1)	265 (16.0)
Active	1442	313 (21.7)	601 (41.7)	195 (13.5)	139 (9.6)	194 (13.5)
**Alcohol intake at TP2, units per week**						
Mean ±SD	5.7 ±8.2	6.3 ±8.0	5.8 ±7.9	5.5 ±8.3	5.3 ±7.8	5.5 ±9.1
**Plasma vitamin C at TP2, µmol/L**						
Mean ±SD	63.0 ±22.2	66.0 ±21.5	65.7 ±21.6	63.2 ±22.8	59.9 ±22.4	57.7 ±21.9
**Systolic blood pressure at TP2, mmHg**						
Mean ±SD	136.5 ±17.1	134.7 ±15.9	135.8 ±16.3	138.5 ±18.4	136.6 ±16.8	138.0 ±18.1
**Total cholesterol at TP2, mmol/L**						
Mean ±SD	5.4 ±1.1	5.6 ±1.1	5.5 ±1.1	5.3 ±1.1	5.2 ±1.2	5.0 ±1.2
**Prevalent heart attack at TP2 (n (%))**						
No reported heart attack at TP2	9455	1833 (19.4)	3211 (34.0)	1499 (15.9)	1116 (11.8)	1796 (19.0)
Self-reported heart attack at TP2	359	28 (7.8)	39 (10.9)	46 (12.8)	60 (16.7)	186 (51.8)
**Prevalent stroke at TP2 (n (%))**						
No reported stroke at TP2	9577	1843 (19.2)	3215 (33.6)	1510 (15.8)	1141 (11.9)	1868 (19.5)
Self-reported stroke at TP2	237	18 (7.6)	35 (14.8)	35 (14.8)	35 (14.8)	114 (48.1)
**Prevalent cancer at TP2 (n (%))**						
No reported cancer at TP2	8888	1744 (19.6)	2987 (33.6)	1398 (15.7)	1052 (11.8)	1707 (19.2)
Self-reported cancer at TP2	926	117 (12.6)	263 (28.4)	147 (15.9)	124 (13.4)	275 (29.7)
**Prevalent diabetes at TP2 (n (%))**						
No reported diabetes at TP2	9477	1834 (19.4)	3238 (34.2)	1477 (15.6)	1124 (11.9)	1804 (19.0)
Self-reported diabetes at TP2	337	27 (8.0)	12 (3.6)	68 (20.2)	52 (15.4)	178 (52.8)

For peer review only - http://bmjopen.bmj.com/site/about/guidelines.xhtml

Supplementary Table S2 | Multivariable logistic regression of risk factors for Charlson 5-year and 10-year hospital admissions with multimorbidity at TP2 in 9814 men and women

	Charlson 5-year multimorbidity †, 2009–2014 OR (95% CI)	p value	Charlson 10-year multimorbidity †, 2009–2019 OR (95% CI)	p value
**Model 1**				
Male sex	1.67 (1.46–1.91)	< 0.001	1.72 (1.55–1.91)	< 0.001
Age per 10 years	2.35 (2.16–2.56)	< 0.001	2.36 (2.21–2.53)	< 0.001
Manual social class at baseline	1.02 (0.88–1.18)	0.774	1.11 (0.99–1.24)	0.072
Lower education level at baseline	1.12 (0.96–1.30)	0.154	1.26 (1.12–1.42)	< 0.001
**Model 2**				
Male sex	1.60 (1.39–1.84)	< 0.001	1.65 (1.48–1.84)	< 0.001
Age per 10 years	2.19 (2.00–2.39)	< 0.001	2.22 (2.07–2.38)	< 0.001
Manual social class at baseline	1.01 (0.87–1.18)	0.850	1.11 (0.99–1.24)	0.082
Lower education level at baseline	1.10 (0.94–1.29)	0.215	1.25 (1.11–1.41)	< 0.001
Prevalent CVD	2.25 (1.78–2.81)	< 0.001	2.60 (2.13–3.18)	< 0.001
Prevalent cancer	1.83 (1.50–2.22)	< 0.001	1.61 (1.37–1.90)	< 0.001
Prevalent diabetes	3.96 (3.08–5.08)	< 0.001	3.91 (3.09–4.96)	< 0.001
**Model 3**				
Male sex	1.44 (1.24–1.67)	< 0.001	1.52 (1.35–1.71)	< 0.001
Age per 10 years	2.14 (1.95–2.35)	< 0.001	2.23 (2.07–2.39)	< 0.001
Manual social class at baseline	0.97 (0.83–1.13)	0.692	1.07 (0.95–1.20)	0.287
Lower education level at baseline	1.04 (0.89–1.22)	0.598	1.20 (1.06–1.35)	0.004
Current smoker	1.44 (1.02–1.99)	0.032	1.45 (1.11–1.86)	0.005
BMI>30 kg/m²	1.38 (1.17–1.63)	< 0.001	1.54 (1.35–1.75)	< 0.001
Alcohol intake, units per week	1.00 (0.99–1.01)	0.611	1.00 (0.99–1.01)	0.664
Physically inactive	1.30 (1.12–1.50)	< 0.001	1.13 (1.01–1.26)	0.034
Plasma vitamin C per SD	0.80 (0.74–0.87)	< 0.001	0.83 (0.79–0.88)	< 0.001
Prevalent CVD	2.11 (1.67–2.64)	< 0.001	2.46 (2.01–3.02)	< 0.001
Prevalent cancer	1.81 (1.48–2.20)	< 0.001	1.61 (1.36–1.89)	< 0.001
Prevalent diabetes	3.55 (2.75–4.56)	< 0.001	3.47 (2.74–4.41)	< 0.001
**Model 4**				
Male sex	1.35 (1.15–1.58)	< 0.001	1.41 (1.25–1.60)	< 0.001
Age per 10 years	2.11 (1.92–2.32)	< 0.001	2.20 (2.04–2.37)	< 0.001
Manual social class at baseline	0.97 (0.83–1.13)	0.692	1.07 (0.95–1.20)	0.287
Lower education level at baseline	1.04 (0.89–1.22)	0.609	1.20 (1.06–1.35)	0.004
Current smoker	1.43 (1.02–1.98)	0.034	1.44 (1.10–1.85)	0.006
BMI>30 kg/m²	1.37 (1.16–1.62)	< 0.001	1.53 (1.34–1.74)	< 0.001
Alcohol intake, units per week	1.00 (0.99–1.01)	0.449	1.00 (1.00–1.01)	0.416
Physically inactive	1.29 (1.12–1.50)	< 0.001	1.13 (1.01–1.26)	0.038
Plasma vitamin C per SD	0.81 (0.75–0.87)	< 0.001	0.84 (0.79–0.89)	< 0.001
Systolic blood pressure per SD	0.99 (0.92–1.07)	0.854	0.99 (0.94–1.05)	0.769
Total cholesterol per SD	0.91 (0.84–0.98)	0.014	0.89 (0.84–0.95)	< 0.001
Prevalent CVD	2.02 (1.60–2.54)	< 0.001	2.34 (1.91–2.87)	< 0.001
Prevalent cancer	1.81 (1.48–2.20)	< 0.001	1.60 (1.36–1.89)	< 0.001
Prevalent diabetes	3.28 (2.52–4.24)	< 0.001	3.16 (2.48–4.03)	< 0.001

† Charlson Comorbidity Index ≥3 vs Charlson ≤2 or no hospital admission.

For peer review only

For peer review only - http://bmjopen.bmj.com/site/about/guidelines.xhtml

Supplementary Table S3 | Multivariable logistic regression of risk factors excluding participants with prevalent CVD, cancer or diabetes at TP2 for Charlson 5-year and 10-year hospital admissions with multimorbidity at TP2 in 8185 men and women

	Charlson 5-year multimorbidity †, 2009–2014 OR (95% CI)	p value	Charlson 10-year multimorbidity †, 2009–2019 OR (95% CI)	p value
**Model 1**				
Male sex	1.44 (1.22–1.70)	< 0.001	1.55 (1.37–1.75)	< 0.001
Age per 10 years	2.36 (2.13–2.62)	< 0.001	2.39 (2.21–2.58)	< 0.001
Manual social class at baseline	0.99 (0.82–1.18)	0.888	1.11 (0.97–1.27)	0.122
Lower education level at baseline	1.12 (0.93–1.36)	0.220	1.26 (1.09–1.44)	0.001
**Model 2**				
Male sex	1.25 (1.05–1.50)	0.015	1.39 (1.22–1.59)	< 0.001
Age per 10 years	2.36 (2.12–2.63)	< 0.001	2.43 (2.24–2.64)	< 0.001
Manual social class at baseline	0.93 (0.78–1.12)	0.471	1.06 (0.93–1.22)	0.373
Lower education level at baseline	1.06 (0.88–1.29)	0.518	1.21 (1.05–1.39)	0.008
Current smoker	1.81 (1.23–2.59)	0.002	1.66 (1.24–2.20)	< 0.001
BMI>30 kg/m²	1.42 (1.16–1.73)	< 0.001	1.60 (1.38–1.86)	< 0.001
Alcohol intake, units per week	1.01 (1.00–1.02)	0.238	1.01 (1.00–1.01)	0.109
Physically inactive	1.18 (0.99–1.41)	0.061	1.10 (0.97–1.26)	0.138
Plasma vitamin C per SD	0.79 (0.72–0.86)	< 0.001	0.83 (0.77–0.88)	< 0.001
**Model 3**				
Male sex	1.16 (0.96–1.40)	0.135	1.29 (1.12–1.49)	< 0.001
Age per 10 years	2.29 (2.05–2.57)	< 0.001	2.39 (2.20–2.60)	< 0.001
Manual social class at baseline	0.93 (0.77–1.12)	0.446	1.06 (0.93–1.21)	0.385
Lower education level at baseline	1.06 (0.88–1.28)	0.534	1.21 (1.05–1.39)	0.009
Current smoker	1.81 (1.23–2.59)	0.002	1.64 (1.23–2.18)	< 0.001
BMI>30 kg/m²	1.39 (1.14–1.70)	0.001	1.59 (1.36–1.84)	< 0.001
Alcohol intake, units per week	1.01 (1.00–1.02)	0.163	1.01 (1.00–1.02)	0.054
Physically inactive	1.18 (0.99–1.40)	0.071	1.10 (0.96–1.25)	0.161
Plasma vitamin C per SD	0.79 (0.72–0.87)	< 0.001	0.83 (0.77–0.89)	< 0.001
Systolic blood pressure per SD	1.03 (0.95–1.12)	0.466	0.99 (0.93–1.06)	0.866
Total cholesterol per SD	0.88 (0.80–0.96)	0.005	0.89 (0.83–0.95)	< 0.001

† Charlson Comorbidity Index ≥3 vs Charlson ≤2 or no hospital admission.

For peer review only - http://bmjopen.bmj.com/site/about/guidelines.xhtml

Supplementary Table S4 | Charlson Comorbidity Index, ICD-10 codes and weighting

	ICD-10 codes
Myocardial infarction	I21.x, I22.x, I25.2
Congestive heart failure	I09.9, I11.0, I13.0, I13.2, I25.5, I42.0, I42.5–I42.9, I43.x, I50.x, P29.0
Peripheral vascular disease	I70.x, I71.x, I73.1, I73.8, I73.9, I77.1, I79.0, I79.2, K55.1, K55.8, K55.9, Z95.8, Z95.9
Cerebrovascular disease	G45.x, G46.x, H34.0, I60.x–I69.x
Dementia	F00.x–F03.x, F05.1, G30.x, G31.1
Chronic pulmonary disease	I27.8, I27.9, J40.x–J47.x, J60.x–J67.x, J68.4, J70.1, J70.3
Rheumatic disease	M05.x, M06.x, M31.5, M32.x–M34.x, M35.1, M35.3, M36.0
Peptic ulcer disease	K25.x–K28.x
Mild liver disease	B18.x, K70.0–K70.3, K70.9, K71.3–K71.5, K71.7, K73.x, K74.x, K76.0, K76.2–K76.4, K76.8, K76.9, Z94.4
Diabetes without chronic complication	E10.0, E10.1, E10.6, E10.8, E10.9, E11.0, E11.1, E11.6, E11.8, E11.9, E12.0, E12.1, E12.6, E12.8, E12.9, E13.0, E13.1, E13.6, E13.8, E13.9, E14.0, E14.1, E14.6, E14.8, E14.9
Diabetes with chronic complication	E10.2–E10.5, E10.7, E11.2–E11.5, E11.7, E12.2–E12.5, E12.7, E13.2–E13.5, E13.7, E14.2–E14.5, E14.7
Hemiplegia or paraplegia	G04.1, G11.4, G80.1, G80.2, G81.x, G82.x, G83.0–G83.4, G83.9
Renal disease	I12.0, I13.1, N03.2–N03.7, N05.2–N05.7, N18.x, N19.x, N25.0, Z49.0–Z49.2, Z94.0, Z99.2
Any malignancy, including lymphoma and leukaemia, except malignant neoplasm of skin	C00.x–C26.x, C30.x–C34.x, C37.x–C41.x, C43.x, C45.x–C58.x, C60.x–C76.x, C81.x–C85.x, C88.x, C90.x–C97.x
Moderate or severe liver disease	I85.0, I85.9, I86.4, I98.2, K70.4, K71.1, K72.1, K72.9, K76.5, K76.6, K76.7
Metastatic solid tumour	C77.x–C80.x
AIDS/HIV	B20.x–B22.x, B24.x

All comorbidities are assigned a weight of 1 except hemiplegia/paraplegia, renal disease, and malignancies (weight=2); moderate/severe liver disease (weight=3); metastatic solid tumour and AIDS/HIV (weight=6). For diseases with two levels of severity (liver disease, diabetes and cancer), the less severe version is assigned weight=0 if the more severe version is also present in a patient. Reproduced from documentation for the 'comorbidity' R package (Gasparini, 2019)

For peer review only

STROBE Statement—Checklist of items that should be included in reports of ***cohort studies***

	Item No	Recommendation	Page No
**Title and abstract**	1	(*a*) Indicate the study's design with a commonly used term in the title or the abstract	1
		(*b*) Provide in the abstract an informative and balanced summary of what was done and what was found	2
**Introduction**			
Background/rationale	2	Explain the scientific background and rationale for the investigation being reported 6	5
Objectives	3	State specific objectives, including any prespecified hypotheses	5
**Methods**			
Study design	4	Present key elements of study design early in the paper	6
Setting	5	Describe the setting, locations, and relevant dates, including periods of recruitment, exposure, follow-up, and data collection	6-7
Participants	6	(*a*) Give the eligibility criteria, and the sources and methods of selection of participants. Describe methods of follow-up	6, Figure 1 - Flow diagram
		(*b*) For matched studies, give matching criteria and number of exposed and unexposed	
Variables	7	Clearly define all outcomes, exposures, predictors, potential confounders, and effect modifiers. Give diagnostic criteria, if applicable	6-7
Data sources/ measurement	8*	For each variable of interest, give sources of data and details of methods of assessment (measurement). Describe comparability of assessment methods if there is more than one group	6-7
Bias	9	Describe any efforts to address potential sources of bias	
Study size	10	Explain how the study size was arrived at	Figure 1 - Flow diagram
Quantitative variables	11	Explain how quantitative variables were handled in the analyses. If applicable, describe which groupings were chosen and why	8 (statistical methods)
Statistical methods	12	(*a*) Describe all statistical methods, including those used to control for confounding	8 (statistical methods)
		(*b*) Describe any methods used to examine subgroups and interactions	

For peer review only - http://bmjopen.bmj.com/site/about/guidelines.xhtml

		(*c*) Explain how missing data were addressed	8, statistical methods
		(*d*) If applicable, explain how loss to follow-up was addressed	
		(*e*) Describe any sensitivity analyses	
**Results**			
Participants	13*	(a) Report numbers of individuals at each stage of study—eg numbers potentially eligible, examined for eligibility, confirmed eligible, included in the study, completing follow-up, and analysed	Figure 1 (Flow diagram)
		(b) Give reasons for non-participation at each stage	6, Figure 1 (Flow diagram)
		(c) Consider use of a flow diagram	Figure 1 (Flow diagram)
Descriptive data	14*	(a) Give characteristics of study participants (eg demographic, clinical, social) and information on exposures and potential confounders	8, Table 1, Table 2, Figure 2
		(b) Indicate number of participants with missing data for each variable of interest	8, statistical methods
		(c) Summarise follow-up time (eg, average and total amount)	8, Title
Outcome data	15*	Report numbers of outcome events or summary measures over time	8-9,Table1

For peer review only

For peer review only - http://bmjopen.bmj.com/site/about/guidelines.xhtml

Main results	16	(*a*) Give unadjusted estimates and, if applicable, confounder-adjusted estimates and their precision (eg, 95% confidence interval). Make clear which confounders were adjusted for and why they were included<br><br>(*b*) Report category boundaries when continuous variables were categorized<br><br>(*c*) If relevant, consider translating estimates of relative risk into absolute risk for a meaningful time period	10-11, Table 3, table 4
Other analyses	17	Report other analyses done—eg analyses of subgroups and interactions, and sensitivity analyses	8, Supplementary Table 1-4
**Discussion**			
Key results	18	Summarise key results with reference to study objectives	13
Limitations	19	Discuss limitations of the study, taking into account sources of potential bias or imprecision. Discuss both direction and magnitude of any potential bias	13,14
Interpretation	20	Give a cautious overall interpretation of results considering objectives, limitations, multiplicity of analyses, results from similar studies, and other relevant evidence	13,16
Generalisability	21	Discuss the generalisability (external validity) of the study results	15
**Other information**			
Funding	22	Give the source of funding and the role of the funders for the present study and, if applicable, for the original study on which the present article is based	

*Give information separately for exposed and unexposed groups.

Note: An Explanation and Elaboration article discusses each checklist item and gives methodological background and published examples of transparent reporting. The STROBE checklist is best used in conjunction with this article (freely available on the Web sites of PLoS Medicine at http://www.plosmedicine.org/, Annals of Internal Medicine at http://www.annals.org/, and Epidemiology at http://www.epidem.com/). Information on the STROBE Initiative is available at http://www.strobe-statement.org.

For peer review only

Sociodemographic and lifestyle predictors of incident hospital admissions with multimorbidity in a general population 1999–2019: the EPIC-Norfolk cohort

Journal:	*BMJ Open*
Manuscript ID	bmjopen-2020-042115.R1
Article Type:	Original research
Date Submitted by the Author:	04-Aug-2020
Complete List of Authors:	Luben, Robert; University of Cambridge School of Clinical Medicine, Department of Public Health and Primary Care<br>Hayat, Shabina; University of Cambridge School of Clinical Medicine, Department of Public Health and Primary Care<br>Wareham, Nicholas; University of Cambridge School of Clinical Medicine, MRC Epidemiology Unit<br>Pharoah, Paul; University of Cambridge School of Clinical Medicine, Department of Public Health and Primary Care<br>Khaw, Kay-Tee; University of Cambridge School of Clinical Medicine, MRC Epidemiology Unit
**Primary Subject Heading**:	Epidemiology
Secondary Subject Heading:	Health services research, Health policy
Keywords:	EPIDEMIOLOGY, Health policy < HEALTH SERVICES ADMINISTRATION & MANAGEMENT, SOCIAL MEDICINE

SCHOLARONE™ Manuscripts

I, the Submitting Author has the right to grant and does grant on behalf of all authors of the Work (as defined in the below author licence), an exclusive licence and/or a non-exclusive licence for contributions from authors who are: i) UK Crown employees; ii) where BMJ has agreed a CC-BY licence shall apply, and/or iii) in accordance with the terms applicable for US Federal Government officers or employees acting as part of their official duties; on a worldwide, perpetual, irrevocable, royalty-free basis to BMJ Publishing Group Ltd ("BMJ") its licensees and where the relevant Journal is co-owned by BMJ to the co-owners of the Journal, to publish the Work in this journal and any other BMJ products and to exploit all rights, as set out in our licence.

The Submitting Author accepts and understands that any supply made under these terms is made by BMJ to the Submitting Author unless you are acting as an employee on behalf of your employer or a postgraduate student of an affiliated institution which is paying any applicable article publishing charge ("APC") for Open Access articles. Where the Submitting Author wishes to make the Work available on an Open Access basis (and intends to pay the relevant APC), the terms of reuse of such Open Access shall be governed by a Creative Commons licence – details of these licences and which Creative Commons licence will apply to this Work are set out in our licence referred to above.

Other than as permitted in any relevant BMJ Author's Self Archiving Policies, I confirm this Work has not been accepted for publication elsewhere, is not being considered for publication elsewhere and does not duplicate material already published. I confirm all authors consent to publication of this Work and authorise the granting of this licence.

For peer review only

Sociodemographic and lifestyle predictors of incident hospital admissions with multimorbidity in a general population 1999–2019: the EPIC-Norfolk cohort

Robert Luben[1], Shabina Hayat[1], Nicholas Wareham[2], Paul Pharoah[1], Kay-Tee Khaw[1]

1 Department of Public Health and Primary Care, Institute of Public Health, University of Cambridge, Cambridge, United Kingdom
2 MRC Epidemiology Unit, University of Cambridge School of Clinical Medicine, Cambridge United Kingdom
Correspondence to: Robert Luben, Department of Public Health and Primary Care, Strangeways Research Laboratory, Worts Causeway, Cambridge, UK. Email: robert.luben@phpc.cam.ac.uk Telephone: +44 (0)1223 748691

July 29, 2020

For peer review only

Abstract

Background

The ageing population and prevalence of long-term disorders with multimorbidity is a major health challenge worldwide. The associations between comorbid conditions and mortality risk are well established; however, few prospective community-based studies have reported on prior risk factors for incident hospital admissions with multimorbidity. We aimed to explore the independent associations for a range of demographic, lifestyle and physiological determinants and the likelihood of subsequent hospital incident multimorbidity.

Methods

We examined incident hospital admissions with multimorbidity in 25014 men and women aged 40–79 in a British prospective population-based study recruited 1993–1997 and followed-up until 2019. The determinants of incident multimorbidity, defined as Charlson Comorbidity Index ≥3, were investigated using multivariable logistic regression models for the 10-year period 1999–2009 and repeated with independent measurements in a second 10-year period 2009–2019.

Results

Between 1999–2009 18179 participants (73% of the population) had a hospital admission. Baseline 5-year and 10-year incident multimorbidity were observed in 6% and 12% of participants respectively. Age per 10-year increase OR 2.19 (95%CI 2.06–2.33) and male sex OR 1.32 (95%CI 1.19–1.47) predicted incident multimorbidity over 10 years. In the subset free of the most serious diseases at baseline, current smoking OR 1.74 (95%CI 1.52–2.00), BMI >30 kg/m^2 OR 1.40 (95%CI 1.24–1.58) and physical inactivity OR 1.14 (95%CI 1.04–1.26) were positively associated and plasma vitamin C (a biomarker of plant food intake) per SD increase OR 0.85 (95%CI 0.81–0.89) inversely associated with incident 10-year multimorbidity after multivariable adjustment for age, sex, social class, education, alcohol consumption, systolic blood pressure and cholesterol. Results were similar when re-examined for a further time period 2009–2019.

Conclusion

Age, male sex and potentially modifiable lifestyle behaviours including smoking, body mass index, physical inactivity and low fruit and vegetable intake were associated with increased risk of future incident hospital admissions with multimorbidity.

For peer review only

Article summary

Strengths and limitations of this study

• We examined future hospital admission with multimorbidity using a prospective design and a community-based population.

• The relationship between demographic, lifestyle and physiological factors and subsequent multimorbidity was documented.

• Measurements were made at two time-points: in mainly middle-aged participants 40–79 years and mainly old-aged participants of 48–92 years.

• Participants were followed-up over 20 years allowing several time periods to be examined.

• Restricting the definition of multimorbidity to a subset of chronic conditions means some conditions will not be counted.

For peer review only

Acknowledgements: *The authors would like to thank the participants, general practitioners, and staff of EPIC-Norfolk.*

Contributors: *KTK,NW SH and RL were involved in the conception and design of the study. RL drafted the manuscript, with support from KTK and PP. SH contributed to data interpretation. RL was responsible for external data linkage. SH and RL contributed to data collection and acquisition. All authors read and critically revised the manuscript and approved the final manuscript. RL is the guarantor. The corresponding author attests that all listed authors meet authorship criteria and that no others meeting the criteria have been omitted.*

Funding: *The design and conduct of the EPIC-Norfolk study and collection and management of the data was supported by programme grants from the Medical Research Council UK (G9502233, G0401527) and Cancer Research UK (C864/A8257, C864/A2883).*

Competing interests: *All authors have completed the ICMJE uniform disclosure form at www.icmje.org/coi_disclosure.pdf and report: RL,SH,KTK and NW report grants from MRC and CRUK during the conduct of the study; The sponsors had no role in any of the following: study design, data collection, data analysis, interpretation of data, writing of the article, decision to submit it for publication. All authors are independent of funders and sponsors and had access to all the data. No conflicts of interest were declared by any author (apart from the two grants) and they have no other relationships or activities that could appear to have influenced the submitted work.*

Ethical approval: *The work was approval by the East Norfolk and Waveney NHS research governance committee (2005EC07L) and the Norfolk research ethics committee (05/Q0101/191). All participants gave informed signed consent for study participation including access to medical records.*

Data sharing: *The authors will make the dataset available under a Data Transfer Agreement to any bona fide researcher who wishes to obtain the dataset in order to undertake a replication analysis. Although the dataset is anonymised, the breadth of the data included and the multiplicity of variables that are included in this analysis file as primary variables or confounding factors, means that provision of the dataset to other researchers without a Data Transfer Agreement would constitute a risk. Requests for data sharing/access should be submitted to the EPIC Management Committee (epic-norfolk@mrc-epid.cam.ac.uk)*

Transparency: *The lead author (the manuscript's guarantor) affirms that the manuscript is an honest, accurate, and transparent account of the study being reported; that no important aspects of the study have been omitted; and that any discrepancies from the study as planned (and, if relevant, registered) have been explained.*

Open access: *This is an Open Access article distributed in accordance with the terms of the Creative Commons Attribution (CC BY 4.0) license, which permits others to distribute, remix, adapt and build upon this work, for commercial use, provided the original work is properly cited. See: http://creativecommons.org/licenses/by/4.0/*

Patient and public involvement: *The EPIC-Norfolk Study have an active Participants Advisory Panel which meets quarterly to advise on research protocols, suggest ideas and provide feedback on the research including proposed new studies and collaborations. All participants of the EPIC-Norfolk study are informed about the study through regular newsletters as well as public meetings. Information is also disseminated through local community talks in the Norfolk area and science festivals.*

Exclusive licence: *The Corresponding Author has the right to grant on behalf of all authors and does grant on behalf of all authors, a worldwide licence to the Publishers and its licensees in perpetuity, in all forms, formats and media (whether known now or created in the future), to i) publish, reproduce, distribute, display and store the Contribution, ii) translate the Contribution into other languages, create adaptations, reprints, include within collections and create summaries, extracts and/or, abstracts of the Contribution, iii) create any other derivative work(s) based on the Contribution, iv) to exploit all subsidiary rights in the Contribution, v) the inclusion of electronic links from the Contribution to third party material where-ever it may be located; and, vi) licence any third party to do any or all of the above.*

For peer review only

Introduction

The Academy of Medical Sciences 2018 report highlighted multimorbidity as a global priority for research. Patients with multimorbidity experience reduced wellbeing and quality of life and account for a disproportionately high share of healthcare workload and costs. Management of the rising prevalence of long-term disorders is the main challenge facing health-care systems worldwide. [1–3]

Multimorbidity is commonly defined as the presence of multiple diseases or conditions with a cut-off of two or more conditions [4], however there is no agreed definition or classification system, which makes the existing evidence base difficult to interpret. [1] The term comorbidity predates multimorbidity and was used to predict the effect of additional diseases for those with an index disease of interest. [5–7] The Charlson Comorbidity Index (CCI) [8] was originally created to predict mortality in hospital patients after one year and is defined using a set of 17 chronic diseases, weighted according to the risk of death. The index has been widely used with several authors suggesting extensions or modifications to the original definition [9–14] and it remains a common standard to which other systems are often compared. [15]

The associations between comorbid conditions and mortality are well established. [16–20] However, few studies have examined the determinants of incident multimorbidity rather than its consequences [21–26] since most lack detailed demographic, socioeconomic and physiological measurements in population-based men and women prior to the onset of multimorbid disease with subsequent follow-up. Retrospective hospital-based studies examining multimorbidity lack community-based denominators while general practice-based studies are often cross-sectional or examine mortality in already multimorbid patients. Few studies examine factors that predict the likelihood of multimorbidity rather than factors that predict risk of individual component conditions. The large majority of studies conducted to date are cross-sectional, with few prospective community-based studies able to examine incident multimorbidity from subsequent hospitalisation. [1,24,25] In this study, we examine the independent associations for a range of demographic, lifestyle and physiological determinants and the likelihood of subsequent hospital incident multimorbidity. We use the CCI over 5-year and 10-year time periods and re-examine these associations independently in a subset 12 years after baseline since health care policy and the criteria used for admission may have changed over time. We have previously reported on risk factors for hospitalisation [27–29] but here we explore in more detail hospital admissions with multimorbidity, a measure of both health service and individual burden.

Methods

We used data from the European Prospective Investigation into Cancer in Norfolk cohort (EPIC-Norfolk) . [30,31] 25639 men and women aged 40–79 were recruited from general practices in Norfolk, completed a lifestyle questionnaire and attended a baseline health check from 1993 to 1997. Participants were reapproached approximately 12 years later, aged 48–92 with 9814 completing a second questionnaire and 8049 attending a health check at time-point two (TP2). Figure 1 shows a flow diagram of participant numbers at various stages. The cohort was followed-up until 2019 with annual record linkage to hospital episode data. Since linkage was to national databases and migration of cohort participants was rare, there was almost no loss to follow-up.

For peer review only - http://bmjopen.bmj.com/site/about/guidelines.xhtml

The Charlson Comorbidity Index (CCI) is defined using a set of chronic diseases, each having an associated weight (1, 2, 3 or 6) related to the risk of death. The conditions are myocardial infarction, congestive heart failure, peripheral vascular disease, cerebrovascular disease, dementia, chronic pulmonary disease, rheumatoid disease, peptic ulcer disease, liver disease, diabetes, hemiplegia or paraplegia, renal disease, cancer and AIDS/HIV. Two levels of severity are defined for liver disease, diabetes and cancer (details are shown in supplementary table S1). All comorbidities are assigned a weight of 1 except hemiplegia/paraplegia, renal disease, and malignancies (weight=2); moderate/severe liver disease (weight=3); metastatic solid tumour and AIDS/HIV (weight=6). For diseases with two levels of severity (liver disease, diabetes and cancer), the less severe version is assigned weight=0 if the more severe version is also present in a patient. The CCI diseases were assigned diagnosis codes using the International Classification of Diseases (ICD-10) which was used to link the CCI to hospital episode statistics (HES) records and to cohort participants. The weighted individual disease scores were totalled to create an overall score with a maximum value of 29. [8,12] CCI was measured for various outcome periods restricted to all hospital events within the given time period: at baseline, 5-year (1999–2004) and 10-year (1999–2009) CCI and at TP2, 5-year (2009–2014) and 10-year (2009–2019) CCI. Multiple admissions including the same CCI category were only counted once.

Participants attending the baseline and TP2 health examinations had their height to the nearest 0.1 cm measured using a stadiometer (Chasemores, UK) and their weight to the nearest 100g measured in light clothing without shoes (Salter, West Bromwich, UK). Body mass index (BMI) was calculated using measured weight in kilograms divided by the square of measured height in square metres. Trained nurses obtained non-fasting blood samples by venepuncture into plain and citrate bottles. Bloods were assayed at the Department of Clinical Biochemistry, University of Cambridge, UK. Serum concentrations of total cholesterol were measured with the RA 1000 Technicon analyser (Bayer Diagnostics, Basingstoke). Plasma was stabilised in a standardised volume of metaphosphoric acid stored at −70 °C and vitamin C concentrations measured using a fluorometric assay within one week. [32] Systolic blood pressure was measured using an Accutorr Sphygmomanometer (Datascope Medical, Huntington, United Kingdom). Participants sat for three minutes before two measurements were taken with the arm horizontal and held at mid-sternum level. Systolic blood pressure was defined as the average of the two measurements.

At baseline and again at TP2, participants completed a lifestyle questionnaire. Two yes/no questions were used to derive smoking status: “Have you ever smoked as much as one cigarette a day for as long as a year?” and, where a positive response was given, “Do you smoke cigarettes now?” Participants also completed questions about their employment and that of their partner with details of both current and past employment recorded. Occupational social class was defined according to the Registrar General’s classification. [33,34] A list of common UK qualifications was used to establish educational attainment and participants were asked to mark all relevant qualifications. These were then categorised using the highest qualification attained. Participants were asked about their occupational and leisure physical activity. A combined score was created combining leisure and occupational elements and divided into four ordered categories with those who did not complete the question placed in the inactive category. The score was validated against energy expenditure measured by free-living heart rate monitoring with individual

For peer review only - http://bmjopen.bmj.com/site/about/guidelines.xhtml

calibration. [35,36] Participants were asked “Are you a non-drinker/teetotaller now?” and “At present, about how many alcoholic drinks do you have each week” for various types of alcohol. Current units were calculated from the questionnaire responses with one unit equal to a half pint of beer, one glass of wine or fortified wine or a single measure of spirits. Prevalent disease was established from the question “Has the doctor ever told you that you have any of the following?” followed by a list of common conditions including “Heart attack (myocardial infarction)”, “Stroke”, “Cancer” and “Diabetes”.

Statistical methods

Associations were examined both including and excluding chronic disease at baseline and repeated with independent measurements at TP2 in a subset of participants using a second baseline 12 years approximately after the first. The baseline analysis excludes 625 men and women who died before 1999 while at TP2 a further 126 participants who died prior to 2009 were excluded. Dichotomous variables were created for the social class (manual and non-manual), educational attainment (high and low) at baseline and body mass index (>30 and ≤30 kg/m^2) and usual physical activity (active and inactive) at both baseline and TP2. For social class, professional, managerial and technical and non-manual skilled occupations were classed as non-manual while manual skilled, partly skilled and unskilled were classed as manual. For educational attainment, those with qualifications at secondary level or above where classed as high and those with no qualification as low. Hospital outcomes were categorised into five groups, “No hospital admissions”, CCI=0, CCI=1, CCI=2 and CCI ≥3 and hospital admissions with multimorbidity (incident multimorbidity) defined as CCI ≥3. Multivariable logistic regression was used for all models and compared multimorbid participants (CCI ≥3) with those having CCI ≤2 or no hospital admissions. A sensitivity analysis, using identical models to those in the primary analyses for the period 1999–2009, but excluding 80 participants defined as multimorbid having only one condition with a CCI weighting ≥3, gave virtually identical results (results not shown).

The numbers of individuals with missing values for covariables at baseline were: 53 BMI, 218 smoking status, 545 social class, 18 education level. The physical activity score has no missing values since those with missing data were classified as being inactive. Multiple imputation was used to estimate missing values at TP2 most apparent when participants completed questionnaires but did not attend a health examination n=1891. Predictive mean matching with 5 multiple imputations and 50 iterations was used with baseline and TP2 variables. All analyses were performed using the R statistical language (R Foundation for Statistical Computing, Vienna, Austria V3.5.3 with packages knitr, Gmisc, ggplot2, tidyverse, intubate, mice.) CCIs were calculated using the R package “comorbidity”. [37]

Results

Table 1 shows future 5-year and 10-year Charlson Comorbidity Index hospital admission rates from baseline for n=25014 and from time-point two (TP2) for n=9814 according to demographic characteristics in the study population. Between 1999–2009 18179 participants (73% of the population) had a hospital admission. Baseline 5-year and 10-year incident multimorbidity (CCI ≥3) were observed in 6% and 12% of participants respectively. Figure 2 shows 10-year multimorbidity rates by age group and sex. More men had CCI ≥3 than women and those aged >75 years had the highest proportion of admissions with multimorbid

For peer review only

conditions with 14.5% at 5 years and 28.8% at 10 years. Multimorbidity rates at TP2 were slightly higher than baseline with 5-year and 10-year incident CCI ≥3 observed in 10% and 20% of participants respectively and the highest proportion in those >75 years.

Table 1 | Charlson Comorbidity Index hospital admission rates by age-group and sex in men and women aged 40–79, 1999–2019

	Total	No admissions	CCI=0	CCI=1	CCI=2	CCI≥3
**Baseline 5-year follow-up period, 1999–2004 (n(%))**						
Men	11228	5457 (48.6)	3340 (29.7)	988 (8.8)	662 (5.9)	781 (7.0)
Women	13786	7153 (51.9)	4398 (31.9)	953 (6.9)	643 (4.7)	639 (4.6)
≤55 years	9567	6009 (62.8)	2720 (28.4)	411 (4.3)	236 (2.5)	191 (2.0)
(55–65] years	7805	3940 (50.5)	2479 (31.8)	583 (7.5)	408 (5.2)	395 (5.1)
(65–75] years	6933	2489 (35.9)	2322 (33.5)	830 (12.0)	561 (8.1)	731 (10.5)
>75 years	709	172 (24.3)	217 (30.6)	117 (16.5)	100 (14.1)	103 (14.5)
**Baseline 10-year follow-up period, 1999–2009 (n(%))**						
Men	11228	2928 (26.1)	4151 (37.0)	1434 (12.8)	1056 (9.4)	1659 (14.8)
Women	13786	3907 (28.3)	5767 (41.8)	1601 (11.6)	1137 (8.2)	1374 (10.0)
≤55 years	9567	3720 (38.9)	4201 (43.9)	746 (7.8)	476 (5.0)	424 (4.4)
(55–65] years	7805	1973 (25.3)	3259 (41.8)	994 (12.7)	711 (9.1)	868 (11.1)
(65–75] years	6933	1059 (15.3)	2294 (33.1)	1168 (16.8)	875 (12.6)	1537 (22.2)
>75 years	709	83 (11.7)	164 (23.1)	127 (17.9)	131 (18.5)	204 (28.8)
**Time-point two 5-year follow-up period, 2009–2014 (n(%))**						
Men	4252	1428 (33.6)	1355 (31.9)	522 (12.3)	389 (9.1)	558 (13.1)
Women	5562	2234 (40.2)	1793 (32.2)	686 (12.3)	403 (7.2)	446 (8.0)
≤55 years	342	215 (62.9)	92 (26.9)	19 (5.6)	10 (2.9)	6 (1.8)
(55–65] years	3090	1540 (49.8)	1006 (32.6)	277 (9.0)	143 (4.6)	124 (4.0)
(65–75] years	3695	1303 (35.3)	1301 (35.2)	464 (12.6)	286 (7.7)	341 (9.2)
>75 years	2687	604 (22.5)	749 (27.9)	448 (16.7)	353 (13.1)	533 (19.8)
**Time-point two 10-year follow-up period, 2009–2019 (n(%))**						
Men	4252	695 (16.3)	1294 (30.4)	631 (14.8)	558 (13.1)	1074 (25.3)
Women	5562	1166 (21.0)	1956 (35.2)	914 (16.4)	618 (11.1)	908 (16.3)
≤55 years	342	154 (45.0)	122 (35.7)	37 (10.8)	14 (4.1)	15 (4.4)
(55–65] years	3090	905 (29.3)	1241 (40.2)	407 (13.2)	267 (8.6)	270 (8.7)
(65–75] years	3695	589 (15.9)	1309 (35.4)	611 (16.5)	473 (12.8)	713 (19.3)
>75 years	2687	213 (7.9)	578 (21.5)	490 (18.2)	422 (15.7)	984 (36.6)

Descriptive characteristics of the cohort according to 10-year CCI are shown in table 2. Participants with higher number of total admissions and longer duration of hospital stay had higher CCI, with mean duration 58 days and 13 admissions for participants with CCI ≥3 during the 10-year period. Participants with multimorbidity admissions were more likely at baseline examination to be current smokers, less physically active, have higher body mass index (BMI) and have lower plasma vitamin C (a proxy for a diet rich in fruit and vegetables) and report various prevalent conditions.

For peer review only - http://bmjopen.bmj.com/site/about/guidelines.xhtml

Table 2 | Descriptive characteristics at baseline in 25014 men and women aged 40–79 by 10-year Charlson Comorbidity Index, 1999–2009

	Total	No admissions	CCI=0	CCI=1	CCI=2	CCI≥3
**Hospital duration 1999–2009, days**						
Mean ±SD	16.3 ±46.5	0.0 ±0.0	9.1 ±28.3	24.9 ±71.5	30.4 ±43.0	57.8 ±77.5
**Total hospital admissions 1999–2009**						
Mean ±SD	3.8 ±16.2	0.0 ±0.0	2.8 ±3.1	4.5 ±6.0	6.4 ±8.3	13.4 ±43.9
**Age, years**						
Mean ±SD	59.0 ±9.3	55.4 ±8.6	57.9 ±8.8	62.0 ±8.8	62.9 ±8.8	65.0 ±8.0
**Body mass index, kg/m²**						
Mean ±SD	26.4 ±3.9	25.9 ±3.7	26.2 ±3.8	26.8 ±4.1	26.8 ±4.3	27.3 ±4.2
**Cigarette smoking (n (%))**						
Current	2904	751 (25.9)	1008 (34.7)	410 (14.1)	291 (10.0)	444 (15.3)
Former	10423	2558 (24.5)	4007 (38.4)	1352 (13.0)	979 (9.4)	1527 (14.7)
Never	11469	3476 (30.3)	4821 (42.0)	1245 (10.9)	903 (7.9)	1024 (8.9)
**Social class dichotomised (n (%))**						
Non-manual	14717	4400 (29.9)	5707 (38.8)	1733 (11.8)	1256 (8.5)	1621 (11.0)
Manual	9752	2304 (23.6)	4029 (41.3)	1214 (12.4)	886 (9.1)	1319 (13.5)
**Level of education (n (%))**						
Higher level	15866	4922 (31.0)	6333 (39.9)	1724 (10.9)	1277 (8.0)	1610 (10.1)
Lower level	9130	1910 (20.9)	3576 (39.2)	1310 (14.3)	916 (10.0)	1418 (15.5)
**Simple physical activity index (n (%))**						
Inactive	7559	1681 (22.2)	2666 (35.3)	1116 (14.8)	788 (10.4)	1308 (17.3)
Moderately inactive	7187	2084 (29.0)	2904 (40.4)	819 (11.4)	610 (8.5)	770 (10.7)
Moderately active	5688	1708 (30.0)	2353 (41.4)	608 (10.7)	470 (8.3)	549 (9.7)
Active	4580	1362 (29.7)	1995 (43.6)	492 (10.7)	325 (7.1)	406 (8.9)
**Alcohol intake, units per week**						
Mean ±SD	7.1 ±9.5	7.7 ±9.6	6.9 ±9.1	6.9 ±9.5	6.7 ±9.8	6.8 ±10.3
**Plasma vitamin C, µmol/L**						
Mean ±SD	53.5 ±20.3	55.3 ±19.8	55.4 ±19.9	50.5 ±20.3	51.3 ±20.9	47.6 ±20.6
**Systolic blood pressure, mmHg**						
Mean ±SD	135.3 ±18.3	132.4 ±17.4	133.5 ±17.5	138.7 ±18.7	138.6 ±19.2	142.2 ±19.3
**Total cholesterol, mmol/L**						
Mean ±SD	6.2 ±1.2	6.1 ±1.1	6.1 ±1.1	6.3 ±1.2	6.2 ±1.2	6.3 ±1.2
**Prevalent heart attack (n (%))**						
No reported heart attack	24253	6745 (27.8)	9764 (40.3)	2886 (11.9)	2097 (8.6)	2761 (11.4)
Self-reported heart attack	728	85 (11.7)	143 (19.6)	146 (20.1)	94 (12.9)	260 (35.7)
**Prevalent stroke (n (%))**						
No reported stroke	24660	6786 (27.5)	9821 (39.8)	2975 (12.1)	2151 (8.7)	2927 (11.9)
Self-reported stroke	329	45 (13.7)	87 (26.4)	57 (17.3)	41 (12.5)	99 (30.1)
**Prevalent cancer (n (%))**						
No reported cancer	23688	6595 (27.8)	9449 (39.9)	2878 (12.1)	2031 (8.6)	2735 (11.5)
Self-reported cancer	1301	237 (18.2)	459 (35.3)	155 (11.9)	162 (12.5)	288 (22.1)
**Prevalent diabetes (n (%))**						
No reported diabetes	24442	6760 (27.7)	9844 (40.3)	2941 (12.0)	2111 (8.6)	2786 (11.4)
Self-reported diabetes	541	71 (13.1)	61 (11.3)	90 (16.6)	81 (15.0)	238 (44.0)

For peer review only

In table 3, odds ratios are shown for 5-year and 10-year incident multimorbidity, defined as those with CCI ≥3, compared with CCI ≤2 or no hospital admission, adjusted age, sex, occupational social class and educational attainment in model 1. Model 2 additionally adjusts for prevalent diseases, cardiovascular disease (CVD), cancer and diabetes; model 3 adds lifestyle factors, current smoking, alcohol units per week, usual physical activity as well as BMI >30 kg/m^2 and plasma vitamin C; model 4 adds systolic blood pressure and cholesterol. Age, sex and prevalent diseases were strongly associated with multimorbidity admissions in all models. The fully adjusted association of 10-year incident multimorbidity with age per 10 years increase was OR 2.19 (95% CI 2.06–2.33), for sex OR 1.32 (95% CI 1.19–1.47), for prevalent CVD OR 2.22 (95% CI 1.87–2.62), cancer OR 2.05 (95% CI 1.73–2.42) and diabetes OR 3.41 (95% CI 2.74–4.24). The risk of multimorbidity in participants with CVD at baseline is equivalent to the risk in those without CVD 10 years older. Similarly, in participants with baseline diabetes and baseline cancer, the risk equivalent to those

For peer review only - http://bmjopen.bmj.com/site/about/guidelines.xhtml

without disease is 17 and 11 years older respectively.

For peer review only

Table 3 | Multivariable logistic regression of risk factors for 5-year and 10-year hospital admissions with multimorbidity in 25014 men and women

	5-year multimorbidity †, 1999–2004 OR (95% CI)	p value	10-year multimorbidity †, 1999–2009 OR (95% CI)	p value
**Model 1**				
Male sex	1.49 (1.34–1.67)	< 0.001	1.56 (1.44–1.69)	< 0.001
Age per 10 years	2.27 (2.13–2.44)	< 0.001	2.34 (2.23–2.46)	< 0.001
Manual social class	1.20 (1.07–1.35)	0.002	1.22 (1.12–1.33)	< 0.001
Lower education level	1.15 (1.02–1.30)	0.023	1.19 (1.09–1.30)	< 0.001
**Model 2**				
Male sex	1.39 (1.24–1.56)	< 0.001	1.47 (1.35–1.60)	< 0.001
Age per 10 years	2.11 (1.97–2.26)	< 0.001	2.21 (2.10–2.32)	< 0.001
Manual social class	1.22 (1.08–1.37)	0.001	1.23 (1.13–1.34)	< 0.001
Lower education level	1.13 (1.00–1.28)	0.053	1.17 (1.07–1.28)	< 0.001
Prevalent CVD	2.23 (1.85–2.68)	< 0.001	2.25 (1.93–2.60)	< 0.001
Prevalent cancer	2.11 (1.75–2.54)	< 0.001	1.92 (1.65–2.22)	< 0.001
Prevalent diabetes	4.41 (3.55–5.45)	< 0.001	4.32 (3.57–5.21)	< 0.001
**Model 3**				
Male sex	1.24 (1.07–1.42)	0.003	1.33 (1.20–1.47)	< 0.001
Age per 10 years	2.16 (1.99–2.34)	< 0.001	2.29 (2.16–2.43)	< 0.001
Manual social class	1.09 (0.95–1.25)	0.214	1.17 (1.06–1.29)	0.002
Lower education level	1.06 (0.92–1.21)	0.447	1.08 (0.98–1.20)	0.112
Current smoker	1.71 (1.42–2.05)	< 0.001	1.73 (1.51–1.98)	< 0.001
BMI>30 kg/m²	1.32 (1.12–1.56)	< 0.001	1.45 (1.28–1.63)	< 0.001
Alcohol intake, units per week	1.00 (0.99–1.01)	0.872	1.00 (1.00–1.01)	0.666
Physically inactive	1.26 (1.10–1.44)	< 0.001	1.15 (1.04–1.26)	0.006
Plasma vitamin C per SD	0.81 (0.75–0.86)	< 0.001	0.84 (0.80–0.88)	< 0.001
Prevalent CVD	2.02 (1.63–2.49)	< 0.001	2.17 (1.84–2.57)	< 0.001
Prevalent cancer	2.22 (1.79–2.72)	< 0.001	2.06 (1.74–2.43)	< 0.001
Prevalent diabetes	3.53 (2.73–4.52)	< 0.001	3.54 (2.85–4.39)	< 0.001
**Model 4**				
Male sex	1.23 (1.07–1.43)	0.005	1.32 (1.19–1.47)	< 0.001
Age per 10 years	2.08 (1.91–2.27)	< 0.001	2.19 (2.06–2.33)	< 0.001
Manual social class	1.09 (0.95–1.25)	0.235	1.16 (1.05–1.28)	0.004
Lower education level	1.06 (0.92–1.22)	0.420	1.09 (0.99–1.21)	0.091
Current smoker	1.72 (1.43–2.07)	< 0.001	1.74 (1.52–2.00)	< 0.001
BMI>30 kg/m²	1.31 (1.11–1.54)	0.001	1.40 (1.24–1.58)	< 0.001
Alcohol intake, units per week	1.00 (0.99–1.01)	0.878	1.00 (1.00–1.01)	0.800
Physically inactive	1.25 (1.09–1.43)	0.001	1.14 (1.04–1.26)	0.008
Plasma vitamin C per SD	0.81 (0.76–0.87)	< 0.001	0.85 (0.81–0.89)	< 0.001
Systolic blood pressure per SD	1.10 (1.03–1.17)	0.005	1.12 (1.07–1.18)	< 0.001
Total cholesterol per SD	0.99 (0.92–1.05)	0.690	0.99 (0.94–1.04)	0.614
Prevalent CVD	2.06 (1.66–2.54)	< 0.001	2.22 (1.87–2.62)	< 0.001
Prevalent cancer	2.23 (1.80–2.75)	< 0.001	2.05 (1.73–2.42)	< 0.001
Prevalent diabetes	3.42 (2.64–4.39)	< 0.001	3.41 (2.74–4.24)	< 0.001

† Charlson Comorbidity Index ≥3 vs Charlson Comorbidity Index ≤2 or no hospital admission.

For peer review only

The models in table 4 are similar to those used in table 3 but rather than adjusting for prevalent disease, participants who reported heart attack, stroke, cancer or diabetes at baseline were excluded. In this subgroup of participants without known common major diseases, in addition to age and sex, current cigarette smoking OR 1.74 (95% CI 1.52–2.00), BMI >30 kg/m² OR 1.40 (95% CI 1.24–1.58) and physical inactivity OR 1.14 (95% CI 1.04–1.26) were positively associated and plasma vitamin C OR 0.85 (95% CI 0.81–0.89) inversely associated with incident 10-year hospital admissions with multimorbidity after multivariable adjustment for age, sex, social class, education, alcohol consumption, systolic blood pressure and cholesterol (model 3). Manual social class and educational attainment were associated with incident multimorbidity in model 1 but were attenuated in model 2 and 3. An inverse association was observed for total cholesterol while systolic blood pressure appeared to be associated but the direction of association was

not consistent with the repeated analyses from TP2. There was no association for alcohol in these models. The risk of multimorbidity in current cigarette smokers is equivalent to the risk in non-smokers 7 years older, while each 20 μmol/L rise in plasma vitamin C (approximately two servings of fruit and vegetables per day [38]) corresponds to a reduction in risk equivalent risk of those 3 years younger.

For peer review only

Table 4 | Multivariable logistic regression of risk factors excluding participants with prevalent CVD, cancer or diabetes for 5-year and 10-year hospital admissions with multimorbidity in 22278 men and women

	5-year multimorbidity †, 1999–2004 OR (95% CI)	p value	10-year multimorbidity †, 1999–2009 OR (95% CI)	p value
**Model 1**				
Male sex	1.47 (1.29–1.68)	< 0.001	1.52 (1.38–1.67)	< 0.001
Age per 10 years	2.19 (2.02–2.37)	< 0.001	2.31 (2.19–2.45)	< 0.001
Manual social class	1.23 (1.07–1.42)	0.003	1.22 (1.11–1.34)	< 0.001
Lower education level	1.20 (1.04–1.39)	0.011	1.16 (1.05–1.28)	0.003
**Model 2**				
Male sex	1.32 (1.13–1.55)	< 0.001	1.39 (1.24–1.55)	< 0.001
Age per 10 years	2.24 (2.05–2.46)	< 0.001	2.40 (2.25–2.56)	< 0.001
Manual social class	1.13 (0.96–1.32)	0.131	1.17 (1.05–1.30)	0.006
Lower education level	1.07 (0.91–1.25)	0.416	1.05 (0.93–1.17)	0.428
Current smoker	1.85 (1.50–2.26)	< 0.001	1.84 (1.58–2.13)	< 0.001
BMI>30 kg/m²	1.31 (1.07–1.58)	0.006	1.53 (1.34–1.75)	< 0.001
Alcohol intake, units per week	1.00 (0.99–1.01)	0.789	1.00 (1.00–1.01)	0.805
Physically inactive	1.25 (1.07–1.46)	0.004	1.17 (1.05–1.31)	0.005
Plasma vitamin C per SD	0.82 (0.76–0.89)	< 0.001	0.85 (0.80–0.90)	< 0.001
**Model 3**				
Male sex	1.32 (1.12–1.56)	0.001	1.37 (1.22–1.54)	< 0.001
Age per 10 years	2.15 (1.95–2.37)	< 0.001	2.30 (2.15–2.46)	< 0.001
Manual social class	1.11 (0.95–1.31)	0.178	1.15 (1.03–1.29)	0.012
Lower education level	1.07 (0.91–1.26)	0.383	1.05 (0.94–1.18)	0.393
Current smoker	1.88 (1.52–2.30)	< 0.001	1.86 (1.60–2.15)	< 0.001
BMI>30 kg/m²	1.30 (1.07–1.58)	0.007	1.48 (1.30–1.70)	< 0.001
Alcohol intake, units per week	1.00 (0.99–1.01)	0.828	1.00 (0.99–1.01)	0.941
Physically inactive	1.24 (1.06–1.45)	0.007	1.16 (1.04–1.29)	0.009
Plasma vitamin C per SD	0.83 (0.77–0.90)	< 0.001	0.86 (0.81–0.91)	< 0.001
Systolic blood pressure per SD	1.12 (1.03–1.21)	0.005	1.13 (1.07–1.19)	< 0.001
Total cholesterol per SD	0.98 (0.91–1.06)	0.607	0.97 (0.92–1.03)	0.328

† Charlson Comorbidity Index ≥3 vs Charlson Comorbidity Index ≤2 or no hospital admission.

Supplementary table S1 shows the ICD-10 codes corresponding to Charlson Comorbidity Index disease groups. Supplementary table S2 shows the descriptive characteristics of participants at TP2 for 10-year CCI. Mean age in this subset, measured approximately 12 years after baseline, was 69.4. Hospital admissions and length of stay was similar to the baseline with multimorbid participants (CCI ≥3) having much longer duration than those with non-multimorbid participants or those who had no hospital admissions. Multimorbid participants were inactive, had lower plasma vitamin C (reflecting a lower intake of fruit and vegetables), were current or former smokers and had prevalent disease. In supplementary table S3, multivariable models of 10-year incident multimorbidity show that prevalent diabetes, CVD and cancer are all strongly associated. After excluding prevalent disease, supplementary table S4 shows multivariable associations in a group free from the most serious diseases at TP2. Both age and male sex are associated with subsequent multimorbidity, with educational attainment, current cigarette smoking, plasma vitamin C, BMI>30 kg/m^2 and physical inactivity all predicting future multimorbidity. Systolic blood pressure was attenuated while other factors including cholesterol were more strongly associated than at baseline.

For peer review only

Discussion

In this community-based population followed prospectively, we observed incident hospital multimorbidity admissions rates over 5-year and 10-years periods which, as expected were strongly related to increasing age. We also observed that those with multimorbid hospital admissions had substantially more days in hospital over the outcome periods. In multivariable analyses, risk of such admissions is predicted by age, male sex and several potentially modifiable factors. Participants at baseline who smoked cigarettes, had BMI >30, were physically inactive or had a diet low in fruit and vegetables all had higher likelihood of having subsequent hospital admissions with multimorbidity. Measurements made on a subset of the cohort 12 years after baseline who were followed up subsequently confirmed the baseline findings while also demonstrating an association for low education level in an older cohort with incident multimorbidity.

Strengths and limitations of study

Most studies of multimorbidity focus on its consequences and those examining risk factors for multimorbidity are largely cross-sectional. While many prospective studies have examined the relationship between baseline characteristics and specific incident diseases or mortality, establishing multimorbidity as an endpoint is more challenging. By using the Charlson Comorbidity Index to define multimorbidity, we were able to show that the chronic diseases defined by the index had considerably higher average length of stay than other conditions requiring hospitalisation and that length of stay increased with higher CCI score. The current population-based study in a defined community was able to assess incident hospital admissions with multimorbidity to enable estimates of 5-year and 10-year rates by age and sex. We were also able to document the relationship between demographic, lifestyle and physiological factors and subsequent hospitalisations for multimorbidity. The EPIC-Norfolk cohort has been followed for 20 years enabling us to examine the determinants of multimorbidity at two time-points: in mainly middle-aged participants of 40–79 years and mainly old-aged participants of 48–92 years in a sub-cohort 12 years later after major organisational changes had been made to the National Health Service (NHS). We were also able to examine associations with and without excluding participants with known prevalent conditions at baseline.

While not attempting to examine clusters or pathways of chronic disease, we have identified risk factors that predict any hospital admissions with multimorbidity. It is possible that some factors we observed will be more strongly associated with certain combinations of diseases and others less so. However, the burden of resources experienced by hospitals can best be mitigated by early public health advice, prior to the onset of disease if possible, which can only be general in nature. Our findings are in line with current public health advice such as smoking cessation, a diet containing fruit and vegetables and regular exercise and, given the huge additional burden placed on the NHS by multimorbidity, should further emphasise the need for public health advice and intervention.

Multimorbidity can be defined in a number of ways such as disease counts or using various indexes. [39] By restricting the definition to a relatively small subset of chronic conditions such as in the CCI, inevitably some conditions will not be counted. It is notable that the CCI does not include depression or mental health, asthma or respiratory diseases, epilepsy, hypothyroidism, musculoskeletal problems or atrial fibrillation, all common in a primary care setting. [40] In addition to the CCI and other commonly used systems [41],

For peer review only

authors have used many other definitions with variable numbers of underlying conditions and hence the prevalence of multimorbidity varies widely. However, CCI is a widely used measure of multimorbidity.

Since the CCI is weighted to predict mortality, it may be better able to assess health service burden than a simple disease count, since procedures required for higher weighted conditions will generally be more costly. However, it may be less effective as an indicator of multiple long-term conditions. Some chronic conditions such as musculoskeletal and mental health diseases not included in the CCI are nevertheless likely to require long stay inpatient care. However, increasing CCI had longer hospital length of stay in the present study and this has also been reported in several other studies. [42,43] Medical conditions such as obesity have well established links to many diseases but, as non-diseases, are not included in the CCI. The use of CCI ≥3 to define multimorbidity classifies a small number of participants with one serious disease with a high CCI weight as multimorbid. However, a sensitivity excluding these people gave virtually identical results. Studies examining the longitudinal predictors of future multimorbidity generally rely on self-reported disease but our study used the CCI from linked hospital medical coding.

When examining the relationship between lifestyle factors and health outcomes, confounding will always be a limitation. Individuals who smoke, are less physically active and eat a poor diet for example, are likely to differ from those with a contrasting lifestyle with respect to other factors relating to the likelihood of future multimorbidity including their age, sex, lifestyle factors examined in this study and others unknown. However, the associations we report were consistent after multivariable adjustment for other factors. Differential mortality is another possible limitation and would occur for any of the factors examined if participants with an apparently unhealthy characteristic were more likely to have died earlier than those with the contrary healthy characteristic and hence were less likely to use hospital services for the full follow-up period. However, the results for the 5-year follow-up period where very few deaths occurred were consistent with the longer 10-year follow-up period. While it is possible that some participants were multimorbid at baseline, we examined those with and without baseline self-reported major chronic disease.

Comparison with other studies

Estimates of the prevalence of multimorbidity vary widely, partly due to the variety of definitions, number of diseases, weighting etc. used in studies but range from 55 to 98% in the elderly .[6] Most studies report multimorbidity associated with age and present in more than half of those aged 65 and older. [3,44] Age was strongly associated with future hospitalisation and incident multimorbidity in our study and has been reported to increase hospitalised multimorbidity in elderly patients. [45] Many studies have found that women have a higher rate of multimorbidity than men [6,44,46–48], but we observed the converse with male sex strongly predicting future multimorbidity. The use of CCI in the context of prospective hospital admissions rather than cross-sectional multimorbidity in a primary care setting may explain the higher proportion of multimorbid men. Physical-mental comorbidity is reported higher among women in primary care [49] and mental health, which is not included in the CCI, may be more likely to be treated in a primary care than in an acute hospital setting.

Despite the considerable literature relating to multimorbidity, very few studies have examined the modifiable determinants of incident multimorbidity. Incident Cancer and cardiometabolic multimorbidity

For peer review only - http://bmjopen.bmj.com/site/about/guidelines.xhtml

was examined in a recent multi-centre study which included data from the present study [21]; pre-diagnostic healthy lifestyle behaviours were reported to be inversely associated with the risk. BMI was also reported to be associated with incident cardiometabolic multimorbidity in a pooled analysis of 16 cohort studies. [22] A Finnish study examined incident multimorbidity in both disease-free and those with baseline diabetes and CVD. [24] They reported some similar findings to the present study such as associations with cigarette smoking, physical inactivity and BMI but associations for low education level and systolic blood pressure were only found in men. Multimorbidity was defined using five common diseases and time to event 10-year follow-up was used rather than a follow-up period approach in this study. Participants in the Finnish cohort were younger than those in EPIC-Norfolk with the oldest participant 74 years at the end of follow-up against 90 years in EPIC-Norfolk baseline and 100 years at TP2. Studies using data from an English longitudinal cohort and using self-reported disease counts to define multimorbidity reported associations in physical activity, obesity and low level of wealth and an increased risk of multimorbidity when combined with other lifestyle factors such smoking, obesity and inadequate fruit and vegetable consumption. [25,26] However, they found no association with educational attainment or excess alcohol consumption. Education, which was associated in older participants at TP2 in our study, has been linked to multimorbidity in cross-sectional studies [50] and prospectively. [24] Socioeconomic status was reported to predict the development of multimorbidity throughout the life course in a Scottish longitudinal study. [51] Both educational attainment and occupational social class were attenuated in our study possibly due to the models including plasma vitamin C, also a marker of socioeconomic status. While smoking was a strong predictor, we did not find an association with alcohol drinking. However, other studies in the literature are inconsistent, with some finding no association with cigarette smoking and alcohol consumption in cross-sectional analyses [1]

Generalisability

While hospital admissions with multimorbidity provide an objective indicator of both Health Service and individual burden of the condition, studies of hospital admissions in many countries are limited by factors relating to differential accessibility to health care such as health insurance, income and health care policy. Though not entirely free of differential accessibility, the NHS in the UK, with service free at the point of delivery for all residents, provides an opportunity to examine hospitalised multimorbidity with fewer of these constraints. Health care policy and criteria for admission change over time, not least in the UK over the 20-year period of this study, so we examined admissions and risk factors for multimorbidity over two independent time periods using new repeated measures and found consistent results.

Conclusions and policy implications

We observed in a long-term population-based study that age, male sex and potentially modifiable factors including smoking, body mass index, physical inactivity and a diet low in fruit and vegetables predict future incident hospitalised multimorbidity. Multimorbidity is increasingly common among elderly hospital inpatients due in part to improved efficacy of treatments and drugs. While considerable effort is being focused on the progression, disease clustering and treatment of patients with multimorbidity, there has been less attention on the long-term predictors of future incident multimorbidity. This study suggests that modest difference in lifestyles may have the potential to mitigate the future burden of multimorbidity in the

For peer review only

population.

For peer review only

References

1 The Academy of Medical Sciences. Multimorbidity: a priority for global health research | The Academy of Medical Sciences. 2018. https://acmedsci.ac.uk/policy/policy-projects/multimorbidity

2 King DE, Xiang J, Pilkerton CS. Multi-morbidity Trends in United States adults, 1988–2014. *J Am Board Fam Med* 2018;**31**:503–13. doi:10.3122/jabfm.2018.04.180008

3 Barnett K, Mercer SW, Norbury M, *et al.* Epidemiology of multimorbidity and implications for health care, research, and medical education: a cross-sectional study. *The Lancet* 2012;**380**:37–43. doi:10.1016/S0140-6736(12)60240-2

4 Johnston MC, Crilly M, Black C, *et al.* Defining and measuring multimorbidity: a systematic review of systematic reviews. *Eur J Public Health* 2019;**29**:182–9. doi:10.1093/eurpub/cky098

5 van den Akker M, Buntinx F, Knottnerus JA. Comorbidity or multimorbidity. *European Journal of General Practice* 1996;**2**:65–70. doi:10.3109/13814789609162146

6 Marengoni A, Angleman S, Melis R, *et al.* Aging with multimorbidity: A systematic review of the literature. *Ageing Research Reviews* 2011;**10**:430–9. doi:10.1016/j.arr.2011.03.003

7 Feinstein AR. THE PRE-THERAPEUTIC CLASSIFICATION OF CO-MORBIDITY IN CHRONIC DISEASE. *J Chronic Dis* 1970;**23**:455–68. doi:10.1016/0021-9681(70)90054-8

8 Charlson ME, Pompei P, Ales KL, *et al.* A new method of classifying prognostic comorbidity in longitudinal studies: Development and validation. *Journal of Chronic Diseases* 1987;**40**:373–83. doi:10.1016/0021-9681(87)90171-8

9 Romano PS, Roos LL, Jollis JG. Presentation adapting a clinical comorbidity index for use with ICD-9-CM administrative data: Differing perspectives. *Journal of Clinical Epidemiology* 1993;**46**:1075–9. doi:10.1016/0895-4356(93)90103-8

10 Groot V de, Beckerman H, Lankhorst GJ, *et al.* How to measure comorbidity: a critical review of available methods. *Journal of Clinical Epidemiology* 2003;**56**:221–9. doi:10.1016/S0895-4356(02)00585-1

11 Extermann M. Measurement and impact of comorbidity in older cancer patients. *Critical Reviews in Oncology/Hematology* 2000;**35**:181–200. doi:10.1016/S1040-8428(00)00090-1

12 Quan H, Sundararajan V, Halfon P, *et al.* Coding Algorithms for Defining Comorbidities in ICD-9-CM and ICD-10 Administrative Data: *Medical Care* 2005;**43**:1130–9. doi:10.1097/01.mlr.0000182534.19832.83

13 Menendez ME, Neuhaus V, van Dijk CN, *et al.* The Elixhauser Comorbidity Method Outperforms the Charlson Index in Predicting Inpatient Death After Orthopaedic Surgery. *Clin Orthop Relat Res* 2014;**472**:2878–86. doi:10.1007/s11999-014-3686-7

14 Quan H, Li B, Couris CM, *et al.* Updating and Validating the Charlson Comorbidity Index and Score for Risk Adjustment in Hospital Discharge Abstracts Using Data From 6 Countries. *Am J Epidemiol* 2011;**173**:676–82. doi:10.1093/aje/kwq433

15 Sharabiani MTA, Aylin P, Bottle A. Systematic Review of Comorbidity Indices for Administrative Data: *Medical Care* 2012;**50**:1109–18. doi:10.1097/MLR.0b013e31825f64d0

16 Caughey GE, Ramsay EN, Vitry AI, *et al.* Comorbid chronic diseases, discordant impact on mortality in older people: a 14-year longitudinal population study. *Journal of Epidemiology & Community Health* 2010;**64**:1036–42. doi:10.1136/jech.2009.088260

17 Lund Jensen N, Pedersen HS, Vestergaard M, *et al.* The impact of socioeconomic status and multimorbidity on mortality: a population-based cohort study. *Clin Epidemiol* 2017;**9**:279–89. doi:10.2147/CLEP.S129415

18 Stanley J, Sarfati D. The new measuring multimorbidity index predicted mortality better than Charlson and Elixhauser indices among the general population. *Journal of Clinical Epidemiology* 2017;**92**:99–110.

For peer review only

doi:10.1016/j.jclinepi.2017.08.005

19 Teh RO, Menzies OH, Connolly MJ, *et al.* Patterns of multi-morbidity and prediction of hospitalisation and all-cause mortality in advanced age. *Age Ageing* 2018;**47**:261–8. doi:10.1093/ageing/afx184

20 Nunes BP, Flores TR, Mielke GI, *et al.* Multimorbidity and mortality in older adults: A systematic review and meta-analysis. *Archives of Gerontology and Geriatrics* 2016;**67**:130–8. doi:10.1016/j.archger.2016.07.008

21 Freisling H, Viallon V, Lennon H, *et al.* Lifestyle factors and risk of multimorbidity of cancer and cardiometabolic diseases: a multinational cohort study. *BMC Medicine* 2020;**18**:5. doi:10.1186/s12916-019-1474-7

22 Kivimäki M, Kuosma E, Ferrie JE, *et al.* Overweight, obesity, and risk of cardiometabolic multimorbidity: pooled analysis of individual-level data for 120 813 adults from 16 cohort studies from the USA and Europe. *The Lancet Public Health* 2017;**2**:e277–85. doi:10.1016/S2468-2667(17)30074-9

23 Gijsen R, Hoeymans N, Schellevis FG, *et al.* Causes and consequences of comorbidity: A review. *Journal of Clinical Epidemiology* 2001;**54**:661–74. doi:10.1016/S0895-4356(00)00363-2

24 Wikström K, Lindström J, Harald K, *et al.* Clinical and lifestyle-related risk factors for incident multimorbidity: 10-year follow-up of Finnish population-based cohorts 1982–2012. *European Journal of Internal Medicine* 2015;**26**:211–6. doi:10.1016/j.ejim.2015.02.012

25 Dhalwani NN, Zaccardi F, O'Donovan G, *et al.* Association Between Lifestyle Factors and the Incidence of Multimorbidity in an Older English Population. *J Gerontol A Biol Sci Med Sci* 2017;**72**:528–34. doi:10.1093/gerona/glw146

26 Mounce LTA, Campbell JL, Henley WE, *et al.* Predicting Incident Multimorbidity. *Ann Fam Med* 2018;**16**:322–9. doi:10.1370/afm.2271

27 Luben R, Hayat S, Wareham N, *et al.* Usual physical activity and subsequent hospital usage over 20 years in a general population: the EPIC-Norfolk cohort. *BMC Geriatrics* 2020;**20**:165. doi:10.1186/s12877-020-01573-0

28 Luben R, Hayat S, Khawaja A, *et al.* Residential area deprivation and risk of subsequent hospital admission in a British population: the EPIC-Norfolk cohort. *BMJ Open* 2019;**9**. doi:10.1136/bmjopen-2019-031251

29 Luben R, Hayat S, Wareham N, *et al.* Predicting admissions and time spent in hospital over a decade in a population-based record linkage study: the EPIC-Norfolk cohort. *BMJ Open* 2016;**6**:e009461. doi:10.1136/bmjopen-2015-009461

30 Day N, Oakes S, Luben R, *et al.* EPIC-Norfolk: study design and characteristics of the cohort. European Prospective Investigation of Cancer. *Br J Cancer* 1999;**80 Suppl 1**:95–103.

31 Hayat SA, Luben R, Keevil VL, *et al.* Cohort Profile: A prospective cohort study of objective physical and cognitive capability and visual health in an ageing population of men and women in Norfolk (EPIC-Norfolk 3). *Int J Epidemiol* 2014;**43**:1063–72. doi:10.1093/ije/dyt086

32 Vuilleumier JP, Keck E. Fluorometric assay of vitamin C in biological materials using a centrifugal analyser with fluorescence attachment. *Journal of micronutrient analysis* Published Online First: 1989.http://agris.fao.org/agris-search/search.do?recordID=US201301738860

33 Bland R. Measuring "Social Class": A Discussion of the Registrar-General's Classification. *Sociology* 1979;**13**:283–91. doi:10.1177/003803857901300209

34 McFadden E, Luben R, Khaw K-T. Different measures of social class in women and mortality. *Eur J Epidemiol* 2009;**24**:231–6. doi:10.1007/s10654-009-9332-0

35 Wareham NJ, Jakes RW, Rennie KL, *et al.* Validity and repeatability of a simple index derived from the short physical activity questionnaire used in the European Prospective Investigation into Cancer and Nutrition (EPIC) study. *Public Health Nutrition* 2003;**6**:407–13. doi:10.1079/PHN2002439

For peer review only

36 Khaw K-T, Jakes R, Bingham S, *et al.* Work and leisure time physical activity assessed using a simple, pragmatic, validated questionnaire and incident cardiovascular disease and all-cause mortality in men and women: The European Prospective Investigation into Cancer in Norfolk prospective population study. *Int J Epidemiol* 2006;**35**:1034–43. doi:10.1093/ije/dyl079

37 Gasparini A. comorbidity: An R package for computing comorbidity scores. *JOSS* 2018;**3**:648. doi:10.21105/joss.00648

38 Khaw K-T, Wareham N, Bingham S, *et al.* Combined Impact of Health Behaviours and Mortality in Men and Women: The EPIC-Norfolk Prospective Population Study. *PLOS Medicine* 2008;**5**:e12. doi:10.1371/journal.pmed.0050012

39 Wallace E, McDowell R, Bennett K, *et al.* Comparison of count-based multimorbidity measures in predicting emergency admission and functional decline in older community-dwelling adults: a prospective cohort study. *BMJ Open* 2016;**6**. doi:10.1136/bmjopen-2016-013089

40 Department of Health UK. Long Term Conditions Compendium of Information: Third Edition. 2012. https://www.gov.uk/government/publications/long-term-conditions-compendium-of-information-third-edition

41 Elixhauser A, Steiner C, Harris DR, *et al.* Comorbidity Measures for Use with Administrative Data: *Medical Care* 1998;**36**:8–27. doi:10.1097/00005650-199801000-00004

42 Librero J, Peiró S, Ordiñana R. Chronic Comorbidity and Outcomes of Hospital Care: Length of Stay, Mortality, and Readmission at 30 and 365 Days. *Journal of Clinical Epidemiology* 1999;**52**:171–9. doi:10.1016/S0895-4356(98)00160-7

43 Palladino R, Tayu Lee J, Ashworth M, *et al.* Associations between multimorbidity, healthcare utilisation and health status: evidence from 16 European countries. *Age Ageing* 2016;**45**:431–5. doi:10.1093/ageing/afw044

44 Nguyen H, Manolova G, Daskalopoulou C, *et al.* Prevalence of multimorbidity in community settings: A systematic review and meta-analysis of observational studies. *J Comorb* 2019;**9**:2235042X1987093. doi:10.1177/2235042X19870934

45 Lai FTT, Wong SYS, Yip BHK, *et al.* Multimorbidity in middle age predicts more subsequent hospital admissions than in older age: A nine-year retrospective cohort study of 121,188 discharged in-patients. *European Journal of Internal Medicine* 2019;**61**:103–11. doi:10.1016/j.ejim.2018.12.001

46 Violán C, Foguet-Boreu Q, Roso-Llorach A, *et al.* Burden of multimorbidity, socioeconomic status and use of health services across stages of life in urban areas: a cross-sectional study. *BMC Public Health* 2014;**14**:530. doi:10.1186/1471-2458-14-530

47 Chung RY, Mercer S, Lai FTT, *et al.* Socioeconomic Determinants of Multimorbidity: A Population-Based Household Survey of Hong Kong Chinese. *PLOS ONE* 2015;**10**:e0140040. doi:10.1371/journal.pone.0140040

48 Alimohammadian M, Majidi A, Yaseri M, *et al.* Multimorbidity as an important issue among women: results of a gender difference investigation in a large population-based cross-sectional study in West Asia. *BMJ Open* 2017;**7**. doi:10.1136/bmjopen-2016-013548

49 Cassell A, Edwards D, Harshfield A, *et al.* The epidemiology of multimorbidity in primary care: a retrospective cohort study. *Br J Gen Pract* 2018;**68**:e245–51. doi:10.3399/bjgp18X695465

50 Nagel G, Peter R, Braig S, *et al.* The impact of education on risk factors and the occurrence of multimorbidity in the EPIC-Heidelberg cohort. *BMC Public Health* 2008;**8**:384. doi:10.1186/1471-2458-8-384

51 Katikireddi SV, Skivington K, Leyland AH, *et al.* The contribution of risk factors to socioeconomic inequalities in multimorbidity across the lifecourse: a longitudinal analysis of the Twenty-07 cohort. *BMC Med* 2017;**15**. doi:10.1186/s12916-017-0913-6

For peer review only

Figure captions

Figure 1 | Flow diagram of cohort recruitment and approaches

Figure 2 | Rate of hospital admissions with multimorbidity, defined as Charlson Comorbidity Index ≥3, by age group and sex, over the 10-year follow-up period 1999–2009

Supplementary material

Supplementary Table S1 | Charlson Comorbidity Index, ICD-10 codes and weighting

Supplementary Table S2 | Descriptive characteristics at TP2 in 9814 men and women aged 48–92 by 10-year Charlson Comorbidity Index, 2009–2019

Supplementary Table S3 | Multivariable logistic regression of risk factors for Charlson 5-year and 10-year hospital admissions with multimorbidity at TP2 in 9814 men and women

Supplementary Table S4 | Multivariable logistic regression of risk factors excluding participants with prevalent CVD, cancer or diabetes at TP2 for Charlson 5-year and 10-year hospital admissions with multimorbidity at TP2 in 8185 men and women

For peer review only

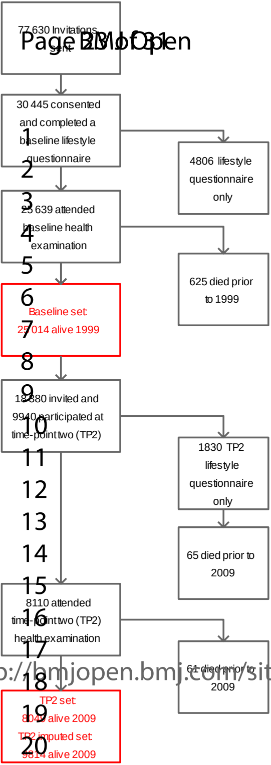

http://bmjopen.bmj.com/sit

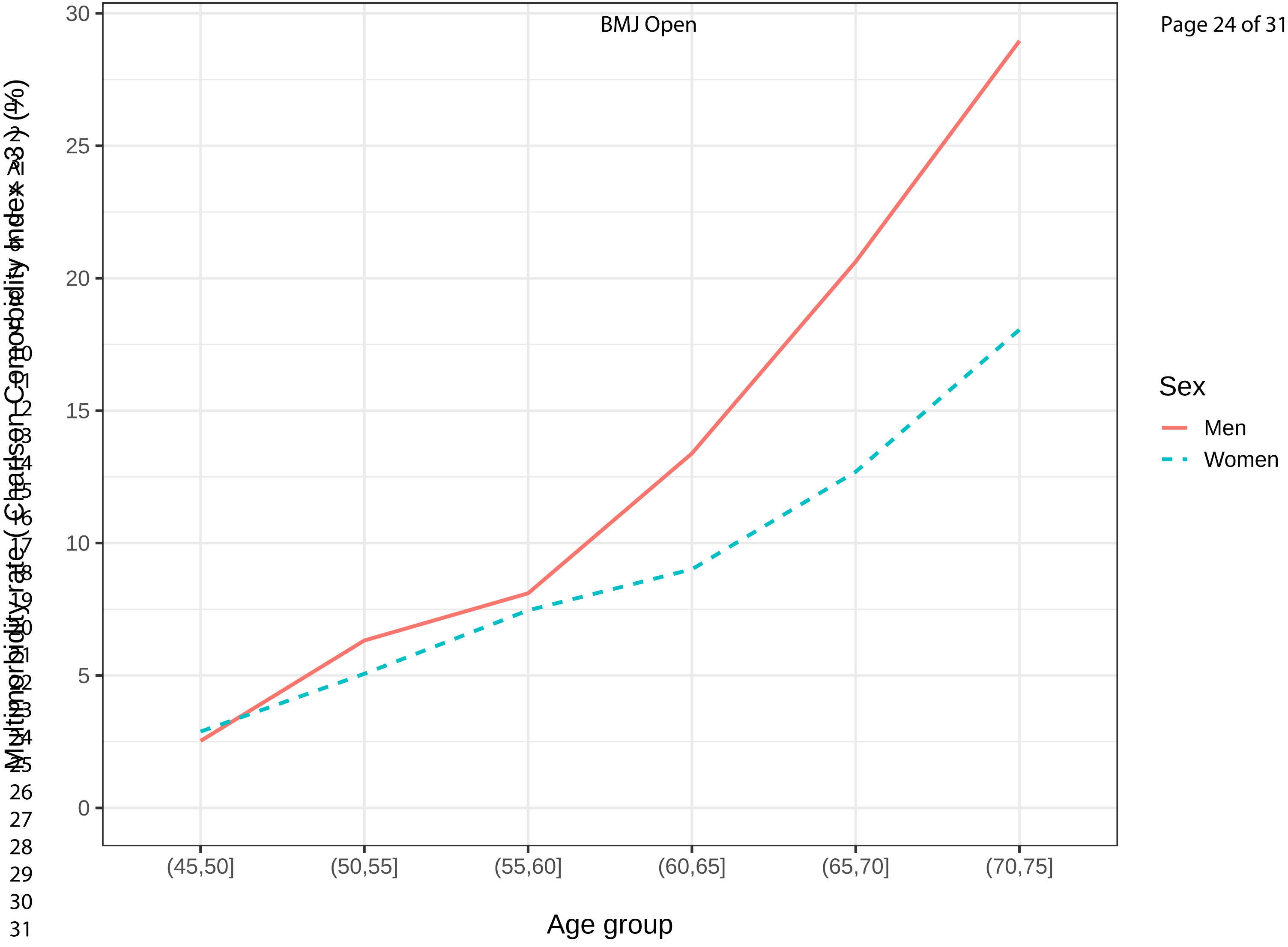
Multimorbidity rate (Charlson Comorbidity Index ≥ 3) (%)
30
25
20
15
10
5
0
(45,50]
(50,55]
(55,60]
(60,65]
(65,70]
(70,75]
Age group
Sex
Men
Women

Sociodemographic and lifestyle predictors of incident hospital admissions with multimorbidity in a general population 1999–2019: the EPIC-Norfolk cohort

Supplementary material

Supplementary Table S1 | Charlson Comorbidity Index, ICD-10 codes and weighting

Supplementary Table S2 | Descriptive characteristics at TP2 in 9814 men and women aged 48–92 by 10-year Charlson Comorbidity Index, 2009–2019

Supplementary Table S3 | Multivariable logistic regression of risk factors for Charlson 5-year and 10-year hospital admissions with multimorbidity at TP2 in 9814 men and women

Supplementary Table S4 | Multivariable logistic regression of risk factors excluding participants with prevalent CVD, cancer or diabetes at TP2 for Charlson 5-year and 10-year hospital admissions with multimorbidity at TP2 in 8185 men and women

Supplementary Table S1 | Charlson Comorbidity Index, ICD-10 codes and weighting

	ICD-10 codes
Myocardial infarction	I21.x, I22.x, I25.2
Congestive heart failure	I09.9, I11.0, I13.0, I13.2, I25.5, I42.0, I42.5–I42.9, I43.x, I50.x, P29.0
Peripheral vascular disease	I70.x, I71.x, I73.1, I73.8, I73.9, I77.1, I79.0, I79.2, K55.1, K55.8, K55.9, Z95.8, Z95.9
Cerebrovascular disease	G45.x, G46.x, H34.0, I60.x–I69.x
Dementia	F00.x–F03.x, F05.1, G30.x, G31.1
Chronic pulmonary disease	I27.8, I27.9, J40.x–J47.x, J60.x–J67.x, J68.4, J70.1, J70.3
Rheumatic disease	M05.x, M06.x, M31.5, M32.x–M34.x, M35.1, M35.3, M36.0
Peptic ulcer disease	K25.x–K28.x
Mild liver disease	B18.x, K70.0–K70.3, K70.9, K71.3–K71.5, K71.7, K73.x, K74.x, K76.0, K76.2–K76.4, K76.8, K76.9, Z94.4
Diabetes without chronic complication	E10.0, E10.1, E10.6, E10.8, E10.9, E11.0, E11.1, E11.6, E11.8, E11.9, E12.0, E12.1, E12.6, E12.8, E12.9, E13.0, E13.1, E13.6, E13.8, E13.9, E14.0, E14.1, E14.6, E14.8, E14.9
Diabetes with chronic complication	E10.2–E10.5, E10.7, E11.2–E11.5, E11.7, E12.2–E12.5, E12.7, E13.2–E13.5, E13.7, E14.2–E14.5, E14.7
Hemiplegia or paraplegia	G04.1, G11.4, G80.1, G80.2, G81.x, G82.x, G83.0–G83.4, G83.9
Renal disease	I12.0, I13.1, N03.2–N03.7, N05.2–N05.7, N18.x, N19.x, N25.0, Z49.0–Z49.2, Z94.0, Z99.2
Any malignancy, including lymphoma and leukaemia, except malignant neoplasm of skin	C00.x–C26.x, C30.x–C34.x, C37.x–C41.x, C43.x, C45.x–C58.x, C60.x–C76.x, C81.x–C85.x, C88.x, C90.x–C97.x
Moderate or severe liver disease	I85.0, I85.9, I86.4, I98.2, K70.4, K71.1, K72.1, K72.9, K76.5, K76.6, K76.7
Metastatic solid tumour	C77.x–C80.x
AIDS/HIV	B20.x–B22.x, B24.x

All comorbidities are assigned a weight of 1 except hemiplegia/paraplegia, renal disease, and malignancies (weight=2); moderate/severe liver disease (weight=3); metastatic solid tumour and AIDS/HIV (weight=6). For diseases with two levels of severity (liver disease, diabetes and cancer), the less severe version is assigned weight=0 if the more severe version is also present in a patient. Reproduced from documentation for the 'comorbidity' R package (Gasparini, 2019)

Supplementary Table S2 | Descriptive characteristics at TP2 in 9814 men and women aged 48–92 by 10-year Charlson Comorbidity Index, 2009–2019

	Total	No admissions	CCI=0	CCI=1	CCI=2	CCI≥3
**Hospital duration 2009–2019, days**						
Mean ±SD	17.7 ±36.2	0.0 ±0.0	6.5 ±14.0	19.3 ±42.8	26.1 ±42.2	46.3 ±48.1
**Total hospital admissions 2009–2019**						
Mean ±SD	4.4 ±7.9	0.0 ±0.0	2.8 ±3.0	4.5 ±6.0	6.0 ±6.7	10.1 ±13.5
**Age at TP2, years**						
Mean ±SD	69.4 ±8.4	65.1 ±7.7	67.6 ±7.7	70.5 ±8.1	71.7 ±8.1	74.1 ±7.8
**Sex (n (%))**						
Men	4252	695 (16.3)	1294 (30.4)	631 (14.8)	558 (13.1)	1074 (25.3)
Women	5562	1166 (21.0)	1956 (35.2)	914 (16.4)	618 (11.1)	908 (16.3)
**Body mass index at TP2, kg/m²**						
Mean ±SD	26.9 ±4.3	26.2 ±4.3	26.5 ±4.0	27.3 ±4.4	27.3 ±4.5	27.7 ±4.5
**Cigarette smoking at TP2 (n (%))**						
Current	442	88 (19.9)	116 (26.2)	65 (14.7)	79 (17.9)	94 (21.3)
Former	4508	755 (16.7)	1375 (30.5)	741 (16.4)	565 (12.5)	1072 (23.8)
Never	4864	1018 (20.9)	1759 (36.2)	739 (15.2)	532 (10.9)	816 (16.8)
**Social class dichotomised (n (%))**						
Non-manual	6294	1205 (19.1)	2127 (33.8)	993 (15.8)	749 (11.9)	1220 (19.4)
Manual	3411	636 (18.6)	1087 (31.9)	528 (15.5)	424 (12.4)	736 (21.6)
**Level of education (n (%))**						
Higher level	7025	1460 (20.8)	2419 (34.4)	1074 (15.3)	791 (11.3)	1281 (18.2)
Lower level	2787	401 (14.4)	830 (29.8)	471 (16.9)	385 (13.8)	700 (25.1)
**Simple physical activity index at TP2 (n (%))**						
Inactive	3924	592 (15.1)	1072 (27.3)	687 (17.5)	545 (13.9)	1028 (26.2)
Moderately inactive	2682	555 (20.7)	940 (35.0)	404 (15.1)	311 (11.6)	472 (17.6)
Moderately active	1654	387 (23.4)	604 (36.5)	231 (14.0)	167 (10.1)	265 (16.0)
Active	1442	313 (21.7)	601 (41.7)	195 (13.5)	139 (9.6)	194 (13.5)
**Alcohol intake at TP2, units per week**						
Mean ±SD	5.7 ±8.2	6.3 ±8.0	5.8 ±7.9	5.5 ±8.3	5.3 ±7.8	5.5 ±9.1
**Plasma vitamin C at TP2, µmol/L**						
Mean ±SD	63.0 ±22.2	66.0 ±21.5	65.7 ±21.6	63.2 ±22.8	59.9 ±22.4	57.7 ±21.9
**Systolic blood pressure at TP2, mmHg**						
Mean ±SD	136.5 ±17.1	134.7 ±15.9	135.8 ±16.3	138.5 ±18.4	136.6 ±16.8	138.0 ±18.1
**Total cholesterol at TP2, mmol/L**						
Mean ±SD	5.4 ±1.1	5.6 ±1.1	5.5 ±1.1	5.3 ±1.1	5.2 ±1.2	5.0 ±1.2
**Prevalent heart attack at TP2 (n (%))**						
No reported heart attack at TP2	9455	1833 (19.4)	3211 (34.0)	1499 (15.9)	1116 (11.8)	1796 (19.0)
Self-reported heart attack at TP2	359	28 (7.8)	39 (10.9)	46 (12.8)	60 (16.7)	186 (51.8)
**Prevalent stroke at TP2 (n (%))**						
No reported stroke at TP2	9577	1843 (19.2)	3215 (33.6)	1510 (15.8)	1141 (11.9)	1868 (19.5)
Self-reported stroke at TP2	237	18 (7.6)	35 (14.8)	35 (14.8)	35 (14.8)	114 (48.1)
**Prevalent cancer at TP2 (n (%))**						
No reported cancer at TP2	8888	1744 (19.6)	2987 (33.6)	1398 (15.7)	1052 (11.8)	1707 (19.2)
Self-reported cancer at TP2	926	117 (12.6)	263 (28.4)	147 (15.9)	124 (13.4)	275 (29.7)
**Prevalent diabetes at TP2 (n (%))**						
No reported diabetes at TP2	9477	1834 (19.4)	3238 (34.2)	1477 (15.6)	1124 (11.9)	1804 (19.0)
Self-reported diabetes at TP2	337	27 (8.0)	12 (3.6)	68 (20.2)	52 (15.4)	178 (52.8)

Supplementary Table S3 | Multivariable logistic regression of risk factors for Charlson 5-year and 10-year hospital admissions with multimorbidity at TP2 in 9814 men and women

	Charlson 5-year multimorbidity †, 2009–2014 OR (95% CI)	p value	Charlson 10-year multimorbidity †, 2009–2019 OR (95% CI)	p value
**Model 1**				
Male sex	1.67 (1.46–1.91)	< 0.001	1.72 (1.55–1.91)	< 0.001
Age per 10 years	2.35 (2.16–2.56)	< 0.001	2.36 (2.21–2.53)	< 0.001
Manual social class at baseline	1.02 (0.88–1.18)	0.774	1.11 (0.99–1.24)	0.072
Lower education level at baseline	1.12 (0.96–1.30)	0.154	1.26 (1.12–1.42)	< 0.001
**Model 2**				
Male sex	1.60 (1.39–1.84)	< 0.001	1.65 (1.48–1.84)	< 0.001
Age per 10 years	2.19 (2.00–2.39)	< 0.001	2.22 (2.07–2.38)	< 0.001
Manual social class at baseline	1.01 (0.87–1.18)	0.850	1.11 (0.99–1.24)	0.082
Lower education level at baseline	1.10 (0.94–1.29)	0.215	1.25 (1.11–1.41)	< 0.001
Prevalent CVD	2.25 (1.78–2.81)	< 0.001	2.60 (2.13–3.18)	< 0.001
Prevalent cancer	1.83 (1.50–2.22)	< 0.001	1.61 (1.37–1.90)	< 0.001
Prevalent diabetes	3.96 (3.08–5.08)	< 0.001	3.91 (3.09–4.96)	< 0.001
**Model 3**				
Male sex	1.44 (1.24–1.67)	< 0.001	1.52 (1.35–1.71)	< 0.001
Age per 10 years	2.14 (1.95–2.35)	< 0.001	2.23 (2.07–2.39)	< 0.001
Manual social class at baseline	0.97 (0.83–1.13)	0.692	1.07 (0.95–1.20)	0.287
Lower education level at baseline	1.04 (0.89–1.22)	0.598	1.20 (1.06–1.35)	0.004
Current smoker	1.44 (1.02–1.99)	0.032	1.45 (1.11–1.86)	0.005
BMI>30 kg/m²	1.38 (1.17–1.63)	< 0.001	1.54 (1.35–1.75)	< 0.001
Alcohol intake, units per week	1.00 (0.99–1.01)	0.611	1.00 (0.99–1.01)	0.664
Physically inactive	1.30 (1.12–1.50)	< 0.001	1.13 (1.01–1.26)	0.034
Plasma vitamin C per SD	0.80 (0.74–0.87)	< 0.001	0.83 (0.79–0.88)	< 0.001
Prevalent CVD	2.11 (1.67–2.64)	< 0.001	2.46 (2.01–3.02)	< 0.001
Prevalent cancer	1.81 (1.48–2.20)	< 0.001	1.61 (1.36–1.89)	< 0.001
Prevalent diabetes	3.55 (2.75–4.56)	< 0.001	3.47 (2.74–4.41)	< 0.001
**Model 4**				
Male sex	1.35 (1.15–1.58)	< 0.001	1.41 (1.25–1.60)	< 0.001
Age per 10 years	2.11 (1.92–2.32)	< 0.001	2.20 (2.04–2.37)	< 0.001
Manual social class at baseline	0.97 (0.83–1.13)	0.692	1.07 (0.95–1.20)	0.287
Lower education level at baseline	1.04 (0.89–1.22)	0.609	1.20 (1.06–1.35)	0.004
Current smoker	1.43 (1.02–1.98)	0.034	1.44 (1.10–1.85)	0.006
BMI>30 kg/m²	1.37 (1.16–1.62)	< 0.001	1.53 (1.34–1.74)	< 0.001
Alcohol intake, units per week	1.00 (0.99–1.01)	0.449	1.00 (1.00–1.01)	0.416
Physically inactive	1.29 (1.12–1.50)	< 0.001	1.13 (1.01–1.26)	0.038
Plasma vitamin C per SD	0.81 (0.75–0.87)	< 0.001	0.84 (0.79–0.89)	< 0.001
Systolic blood pressure per SD	0.99 (0.92–1.07)	0.854	0.99 (0.94–1.05)	0.769
Total cholesterol per SD	0.91 (0.84–0.98)	0.014	0.89 (0.84–0.95)	< 0.001
Prevalent CVD	2.02 (1.60–2.54)	< 0.001	2.34 (1.91–2.87)	< 0.001
Prevalent cancer	1.81 (1.48–2.20)	< 0.001	1.60 (1.36–1.89)	< 0.001
Prevalent diabetes	3.28 (2.52–4.24)	< 0.001	3.16 (2.48–4.03)	< 0.001

† Charlson Comorbidity Index ≥3 vs Charlson Comorbidity Index ≤2 or no hospital admission.

Supplementary Table S4 | Multivariable logistic regression of risk factors excluding participants with prevalent CVD, cancer or diabetes at TP2 for 5-year and 10-year hospital admissions with multimorbidity at TP2 in 8185 men and women

	5-year follow-up period †, 2009–2014 OR (95% CI)	p value	10-year follow-up period †, 2009–2019 OR (95% CI)	p value
**Model 1**				
Male sex	1.44 (1.22–1.70)	< 0.001	1.55 (1.37–1.75)	< 0.001
Age per 10 years	2.36 (2.13–2.62)	< 0.001	2.39 (2.21–2.58)	< 0.001
Manual social class at baseline	0.99 (0.82–1.18)	0.888	1.11 (0.97–1.27)	0.122
Lower education level at baseline	1.12 (0.93–1.36)	0.220	1.26 (1.09–1.44)	0.001
**Model 2**				
Male sex	1.25 (1.05–1.50)	0.015	1.39 (1.22–1.59)	< 0.001
Age per 10 years	2.36 (2.12–2.63)	< 0.001	2.43 (2.24–2.64)	< 0.001
Manual social class at baseline	0.93 (0.78–1.12)	0.471	1.06 (0.93–1.22)	0.373
Lower education level at baseline	1.06 (0.88–1.29)	0.518	1.21 (1.05–1.39)	0.008
Current smoker	1.81 (1.23–2.59)	0.002	1.66 (1.24–2.20)	< 0.001
BMI>30 kg/m²	1.42 (1.16–1.73)	< 0.001	1.60 (1.38–1.86)	< 0.001
Alcohol intake, units per week	1.01 (1.00–1.02)	0.238	1.01 (1.00–1.01)	0.109
Physically inactive	1.18 (0.99–1.41)	0.061	1.10 (0.97–1.26)	0.138
Plasma vitamin C per SD	0.79 (0.72–0.86)	< 0.001	0.83 (0.77–0.88)	< 0.001
**Model 3**				
Male sex	1.16 (0.96–1.40)	0.135	1.29 (1.12–1.49)	< 0.001
Age per 10 years	2.29 (2.05–2.57)	< 0.001	2.39 (2.20–2.60)	< 0.001
Manual social class at baseline	0.93 (0.77–1.12)	0.446	1.06 (0.93–1.21)	0.385
Lower education level at baseline	1.06 (0.88–1.28)	0.534	1.21 (1.05–1.39)	0.009
Current smoker	1.81 (1.23–2.59)	0.002	1.64 (1.23–2.18)	< 0.001
BMI>30 kg/m²	1.39 (1.14–1.70)	0.001	1.59 (1.36–1.84)	< 0.001
Alcohol intake, units per week	1.01 (1.00–1.02)	0.163	1.01 (1.00–1.02)	0.054
Physically inactive	1.18 (0.99–1.40)	0.071	1.10 (0.96–1.25)	0.161
Plasma vitamin C per SD	0.79 (0.72–0.87)	< 0.001	0.83 (0.77–0.89)	< 0.001
Systolic blood pressure per SD	1.03 (0.95–1.12)	0.466	0.99 (0.93–1.06)	0.866
Total cholesterol per SD	0.88 (0.80–0.96)	0.005	0.89 (0.83–0.95)	< 0.001

† Charlson Comorbidity Index ≥3 vs Charlson ≤2 or no hospital admission.

STROBE Statement—Checklist of items that should be included in reports of ***cohort studies***

	Item No	Recommendation	Page No
**Title and abstract**	1	(*a*) Indicate the study's design with a commonly used term in the title or the abstract	1
		(*b*) Provide in the abstract an informative and balanced summary of what was done and what was found	2
**Introduction**			
Background/rationale	2	Explain the scientific background and rationale for the investigation being reported 6	5
Objectives	3	State specific objectives, including any prespecified hypotheses	5
**Methods**			
Study design	4	Present key elements of study design early in the paper	6
Setting	5	Describe the setting, locations, and relevant dates, including periods of recruitment, exposure, follow-up, and data collection	6-7
Participants	6	(*a*) Give the eligibility criteria, and the sources and methods of selection of participants. Describe methods of follow-up	6, Figure 1 - Flow diagram
		(*b*) For matched studies, give matching criteria and number of exposed and unexposed	
Variables	7	Clearly define all outcomes, exposures, predictors, potential confounders, and effect modifiers. Give diagnostic criteria, if applicable	6-7
Data sources/ measurement	8*	For each variable of interest, give sources of data and details of methods of assessment (measurement). Describe comparability of assessment methods if there is more than one group	6-7
Bias	9	Describe any efforts to address potential sources of bias	
Study size	10	Explain how the study size was arrived at	Figure 1 - Flow diagram
Quantitative variables	11	Explain how quantitative variables were handled in the analyses. If applicable, describe which groupings were chosen and why	8 (statistical methods)
Statistical methods	12	(*a*) Describe all statistical methods, including those used to control for confounding	8 (statistical methods)
		(*b*) Describe any methods used to examine subgroups and interactions	

For peer review only

For peer review only - http://bmjopen.bmj.com/site/about/guidelines.xhtml

		(*c*) Explain how missing data were addressed<br>(*d*) If applicable, explain how loss to follow-up was addressed<br>(*e*) Describe any sensitivity analyses	8, statistical methods
**Results**			
Participants	13*	(a) Report numbers of individuals at each stage of study—eg numbers potentially eligible, examined for eligibility, confirmed eligible, included in the study, completing follow-up, and analysed	Figure 1 (Flow diagram)
		(b) Give reasons for non-participation at each stage	6, Figure 1 (Flow diagram)
		(c) Consider use of a flow diagram	Figure 1 (Flow diagram)
Descriptive data	14*	(a) Give characteristics of study participants (eg demographic, clinical, social) and information on exposures and potential confounders	8, Table 1, Table 2, Figure 2
		(b) Indicate number of participants with missing data for each variable of interest	8, statistical methods
		(c) Summarise follow-up time (eg, average and total amount)	8, Title
Outcome data	15*	Report numbers of outcome events or summary measures over time	8-9,Table1

For peer review only

Main results	16	(*a*) Give unadjusted estimates and, if applicable, confounder-adjusted estimates and their precision (eg, 95% confidence interval). Make clear which confounders were adjusted for and why they were included<br><br>(*b*) Report category boundaries when continuous variables were categorized<br><br>(*c*) If relevant, consider translating estimates of relative risk into absolute risk for a meaningful time period	10-11, Table 3, table 4
Other analyses	17	Report other analyses done—eg analyses of subgroups and interactions, and sensitivity analyses	8, Supplementary Table 1-4
**Discussion**			
Key results	18	Summarise key results with reference to study objectives	13
Limitations	19	Discuss limitations of the study, taking into account sources of potential bias or imprecision. Discuss both direction and magnitude of any potential bias	13,14
Interpretation	20	Give a cautious overall interpretation of results considering objectives, limitations, multiplicity of analyses, results from similar studies, and other relevant evidence	13,16
Generalisability	21	Discuss the generalisability (external validity) of the study results	15
**Other information**			
Funding	22	Give the source of funding and the role of the funders for the present study and, if applicable, for the original study on which the present article is based	

*Give information separately for exposed and unexposed groups.

Note: An Explanation and Elaboration article discusses each checklist item and gives methodological background and published examples of transparent reporting. The STROBE checklist is best used in conjunction with this article (freely available on the Web sites of PLoS Medicine at http://www.plosmedicine.org/, Annals of Internal Medicine at http://www.annals.org/, and Epidemiology at http://www.epidem.com/). Information on the STROBE Initiative is available at http://www.strobe-statement.org.

For peer review only
